# Multi-omics subtyping pipeline for chronic obstructive pulmonary disease

**DOI:** 10.1371/journal.pone.0255337

**Published:** 2021-08-25

**Authors:** Lucas A. Gillenwater, Shahab Helmi, Evan Stene, Katherine A. Pratte, Yonghua Zhuang, Ronald P. Schuyler, Leslie Lange, Peter J. Castaldi, Craig P. Hersh, Farnoush Banaei-Kashani, Russell P. Bowler, Katerina J. Kechris

**Affiliations:** 1 National Jewish Health, Denver, CO, United States of America; 2 Department of Computer Science and Engineering, College of Engineering, Design and Computing, University of Colorado Denver, Denver, CO, United States of America; 3 Department of Biostatistics and Informatics, Colorado School of Public Health, University of Colorado Anschutz Medical Campus, Aurora, CO, United States of America; 4 Department of Immunology & Microbiology, University of Colorado, Anschutz Medical Campus, Aurora, CO, United States of America; 5 Division of Biomedical Informatics and Personalized Medicine, Department of Medicine, University of Colorado Anschutz Medical Campus, Aurora, CO, United States of America; 6 Channing Division of Network Medicine, Brigham and Women’s Hospital, Boston, MA, United States of America; Chinese Academy of Sciences, CHINA

## Abstract

Chronic Obstructive Pulmonary Disease (COPD) is the third leading cause of mortality in the United States; however, COPD has heterogeneous clinical phenotypes. This is the first large scale attempt which uses transcriptomics, proteomics, and metabolomics (multi-omics) to determine whether there are molecularly defined clusters with distinct clinical phenotypes that may underlie the clinical heterogeneity. Subjects included 3,278 subjects from the COPDGene cohort with at least one of the following profiles: whole blood transcriptomes (2,650 subjects); plasma proteomes (1,013 subjects); and plasma metabolomes (1,136 subjects). 489 subjects had all three contemporaneous -omics profiles. Autoencoder embeddings were performed individually for each -omics dataset. Embeddings underwent subspace clustering using MineClus, either individually by -omics or combined, followed by recursive feature selection based on Support Vector Machines. Clusters were tested for associations with clinical variables. Optimal single -omics clustering typically resulted in two clusters. Although there was overlap for individual -omics cluster membership, each -omics cluster tended to be defined by unique molecular pathways. For example, prominent molecular features of the metabolome-based clustering included sphingomyelin, while key molecular features of the transcriptome-based clusters were related to immune and bacterial responses. We also found that when we integrated the -omics data at a later stage, we identified subtypes that varied based on age, severity of disease, in addition to diffusing capacity of the lungs for carbon monoxide, and precent on atrial fibrillation. In contrast, when we integrated the -omics data at an earlier stage by treating all data sets equally, there were no clinical differences between subtypes. Similar to clinical clustering, which has revealed multiple heterogenous clinical phenotypes, we show that transcriptomics, proteomics, and metabolomics tend to define clusters of COPD patients with different clinical characteristics. Thus, integrating these different -omics data sets affords additional insight into the molecular nature of COPD and its heterogeneity.

## Introduction

Chronic Obstructive Pulmonary Disease (COPD) is a major cause of morbidity and mortality in the United States, where it affects 22 million Americans and is the 4th leading cause of death [1]. Although >80% of COPD patients are smokers, only about ~20% of smokers develop COPD [2]. Prior research has focused on individual genes or proteins as risk factors, but these candidates typically only account for a small percentage of risk or variance explained with respect to clinical phenotypes. For example, the only well-established genetic risk variant (α1-antitrypsin deficiency) accounts for only 1–2% of COPD cases [3]. Other studies have implicated proteases, oxidative stress, immune defects, and infections as causes of COPD [4, 5]. Furthermore, some smokers develop a predominately emphysema phenotype, characterized by damage to alveoli, where others develop predominantly airway disease.

Because COPD is a heterogeneous disease, the identification of subtypes (i.e., subpopulations of subjects with similar disease characteristics) is of interest and can help increase our understanding of the biologic mechanisms involved in COPD development and progression, and lead to more accurate diagnoses in clinical practice. Subtyping COPD has been performed using clinical data, imaging phenotypes and gene expression in blood [6–8], but not using a multiple -omics approach with appropriate sample size to capture heterogeneity (see smaller studies in [9, 10]).

Limitations for multi-omics subtyping have included small sample size, minimal clinical phenotyping, and sample collection biases. In particular, one of the major barriers to past studies of COPD in humans has been the lack of a large, well-phenotyped cohort of at-risk subjects with corresponding biologic specimens and molecular characterization. The Lung Tissue Research Consortium was started over 5 years ago to overcome this limitation; however, this cohort has limited potential because subjects only included those undergoing surgery, which is not representative of a general population at risk for COPD.

The COPDGene study now has -omics data for a sample size that is much larger than for any previous study of this type (>1,000 participants), providing the unique opportunity to integrate available -omics profiles in an unsupervised learning framework to identify COPD subtypes defined using a variety of molecular features in the blood. This approach offers the advantage of examining consistency across multiple -omics profiles, which should reduce errors since each -omics technology has its own sources of variability, missing data, and technical noise that may lead to classification errors. To identify molecular subtypes for COPD, we conduct dimension reduction using autoencoders, and subspace clustering followed by feature selection on transcriptomic, proteomic and metabolomic data individually, and in an integrated manner using all three data sets.

## Results

Subtyping COPD subjects is important for understanding biological mechanisms and for diagnosis in clinical practice, but has primarily been performed using clinical data, imaging phenotypes, or a single -omics type [6, 8, 11]. Since COPD is a systemic disease and it is difficult and invasive to obtain lung samples, it is also of interest to understand how subjects cluster based on molecular profiles in the blood. This study was possible because we have data from the largest transcriptomic (n = 2,637), proteomic (n = 1,013) and metabolomic studies (n = 1,057) for COPD. Demographic and clinical characteristics of subjects profiled from each study are similar (**Table 1**), however the subjects with proteomics and metabolomics data were somewhat older than the subjects with transcriptomic data (~ 2 years on average, ANOVA p-value < .0001 across all three groups, **Table 1**), more likely to be NHW, have emphysema and airflow obstruction, and only came from two clinical centers (**S1 Table**). A subset of n = 489 subjects was profiled using all three -omics technologies (**S2 Table**).

**Table 1:** Clinical characteristics and demographics for profiled subjects by each of the three -omics technologies.

Variable	Transcriptomic	Proteomic	Metabolomic	p-value^11^
No. of Participants	2637	1013	1057	
Age (mean(sd^1^))	65.5 (8.6)	67.8 (8.6)	67.6 (8.6)	< 0.0001
% Female	48.3	49.3	49.5	0.7668
% AA^2^	25.2	7.9	8.6	< 0.0001
%NHW^2^	74.8	92.1	91.4	
BMI^3^ (mean(sd^1^))	29 (6.3)	29.1 (6.4)	28.9 (6.2)	0.8600
% Former Smoker	64.4	75.3	74.7	< 0.0001
% Current Smoker	35.6	24.7	25.3	
Smoking Pack-Years (mean(sd^1^))	44.1 (23.9)	45.1 (24.8)	45.1 (24.7)	0.3685
% Controls	56.1	52	52	0.0195
% COPD^4^ Cases	43.9	48	48	
% PRISm^5^	12.7	9.3	9.3	0.3346
% GOLD 0	43.4	42.6	42.7	
% GOLD 1	9.9	10.4	10.7	
% GOLD 2	20.1	19.8	20	
% GOLD 3	9.9	11.6	11.3	
% GOLD 4	4.1	6.2	6	
FEV_1_pp^6^ (mean(sd^1^))	78.5 (24.3)	77.2 (26.6)	77.6 (26.4)	0.3563
FEV_1_/FVC^7^ (mean(sd^1^))	0.6762 (0.1471)	0.6572 (0.1549)	0.6581 (0.1544)	0.0002
% Emphysema^8^ (mean(sd^1^))	5.5 (9.3)	7.1 (10.1)	7 (10.1)	< 0.0001
Exacerbation Frequency^9^ (mean(sd^1^))	0.3 (0.8)	0.3 (0.8)	0.3 (0.7)	0.9326
% Chronic Bronchitis^10^	14.7	16.5	16.2	0.443

^1^sd-standard deviations.

^2^NHW—Non-Hispanic White; AA—African American.

^3^BMI–body mass index (kg/m2).

^4^COPD is defined by GOLD score > 0.

^5^PRISm—Preserved Ratio Impaired Spirometry [11].

^6^FEV1/FVC = post-bronchodilator forced expiratory volume at one second (FEV1)/forced vital capacity (FVC)

^7^FEV1pp = FEV_1_ percent predicted.

^8^Quantitative emphysema was quantified by percent of lung voxels -950 Hounsfield Units (% low attenuation areas: %LAA) on the full inspiratory CT scans. Visual emphysema was assessed as described by [12].

^9^Exacerbations were defined as acute worsening of respiratory symptoms requiring treatment with oral corticosteroids and/or antibiotics, emergency room visit, or hospital admission [13].

^10^Chronic bronchitis was defined as self-reported chronic cough and sputum for at least three months in each of the two years prior to baseline.

^11^P- values are reporting for testing the differences of variables across the three different groups of subjects. P-values are based on chi-square tests for categorical or binary variables (sex, smoking status, COPD status, COPD severity by GOLD status, and chronic bronchitis status) or ANOVA tests for continuous variables (age, BMI, smoking pack-years, FEV1pp, FEV1/FVC, percent emphysema, and exacerbation frequency).

### Single-omics analyses

**Fig 1:**
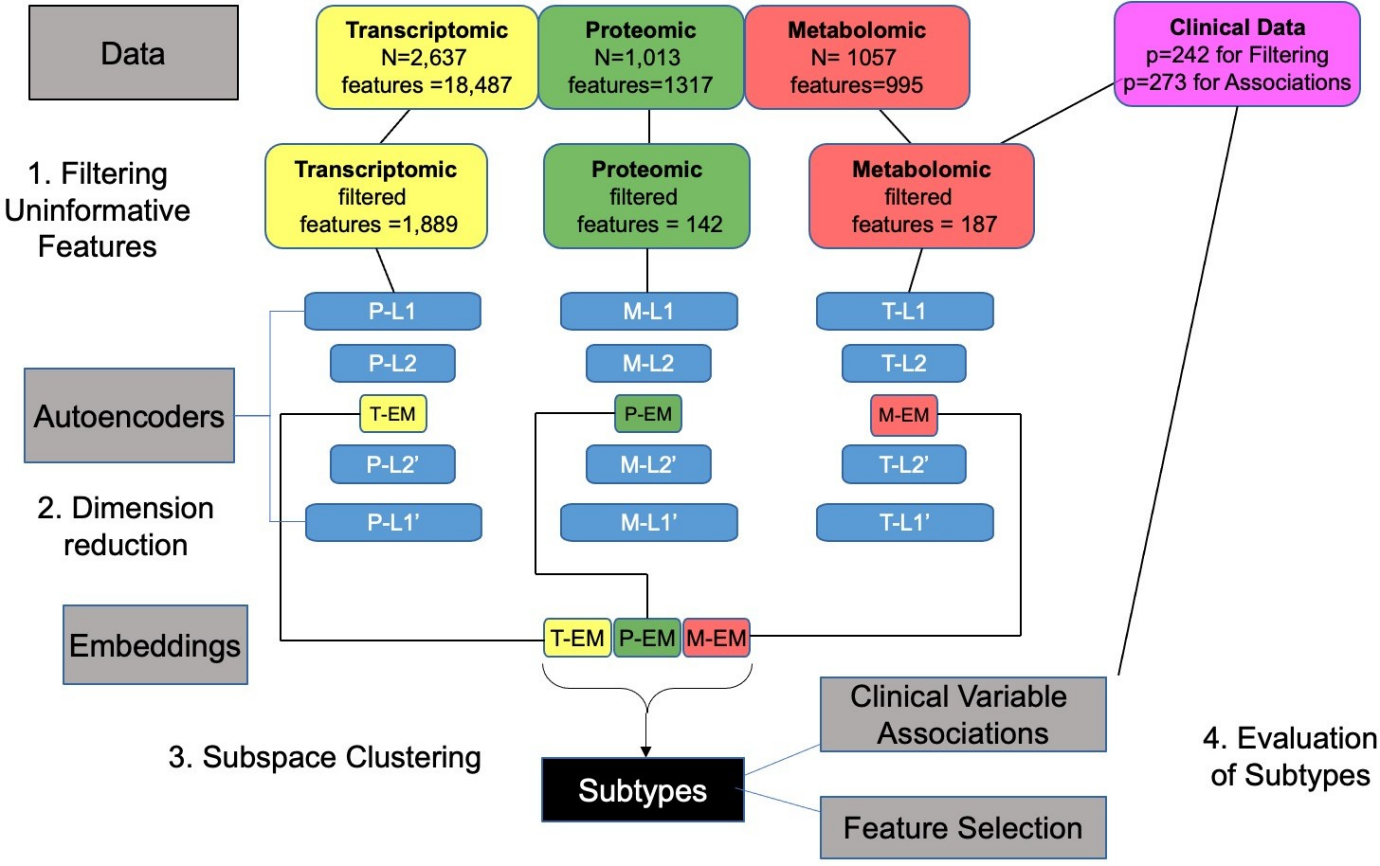
Flow chart illustrating -omics subtyping pipeline. T, P, and M denote the Transcriptomic, Proteomic or Metabolomic data respectively. The sample size (N) and number of features are provided for each of the individual data sets, including the Clinical data. In the first step, uninformative features were filtered by only moving forward with features that had at least one association with demographic, clinical, and imaging variables (but excluding common variables such as age and sex, as well as clinical blood count variables; p = 242 clinical variables). In the second step, dimension reduction was performed using Autoencoders where EM represents embeddings (or using PCA—not shown). In the third step, the reduced dimensions were used to cluster subjects into subtypes using subspace clustering (or k-means—not shown). Finally, the subject subtypes were evaluated in the fourth step by identifying the features (transcripts, proteins, metabolites) or clinical variables (using the complete set p = 273 of clinical variables) that discriminated between the subtypes.

Before embarking on multi-omics integration, we explored the signatures of each -omics profile separately. The overall strategy of our subtyping approach (single -omics and integrated) has four steps (**Fig 1**): 1) covariate filtering, 2) dimensionality reduction 3) clustering and 4) evaluation.

#### Covariate filtering

We devised a covariate filtering approach (see Methods) that was unbiased by systematically testing the association of all molecular features with all demographic, clinical, and imaging variables, and proceeded with only the features that showed at least one association but excluding common variables such as age and sex, as well as clinical blood count variables (**Fig 1, S3 Table**). This filtering method resulted in 1,889 transcripts, 142 proteins and 187 metabolites for clustering. Because of the use of blood samples, we also regressed out cell composition before performing clustering (see Methods).

#### Dimensionality reduction

Even with the reduced number of features, clustering the remaining high-dimensional dataset is problematic due to lack of scalability of clustering methods. To address this problem, we explored dimension reduction methods (see Methods), and focus on the well-established method PCA, and a more recent approached based on deep-learning using auto encoders (AE) [14]. An important tuning parameter for these methods is the number of reduced dimensions (i.e., number of PCs, number of AE embeddings). For both methods, the diagnostic plots based on training and test data (individual and cumulative percent variance explained, see Methods) indicate that eight dimensions is appropriate for all three omics data sets (**S1 Fig in S1 File**). For PCA, eight components explain at least 40% of the variability.

#### Clustering

For most clustering methods, an important tuning parameter is the number of clusters *k*, and the *w* parameter for MineClus, which were selected based on silhouette scores and connectedness (see Methods). We found that *k* = 2 subtypes and using MineClus almost always had higher silhouette and connectedness (**S4 Table**). We found the AE and PC results were similar based on the internal cluster quality metrics, in addition to overlap between subjects (normalized Jaccard similarity > 0.80 for the large subtype discovered by each method). To choose between AE and PC, we examined their ability with eight dimensions to recover the original data as measured by the MSE. AE had lower MSE for all three data sets (0.53, 0.55, 0.54) compared to PC (0.56, 0.66, 0.61) for the transcriptomic, proteomic and metabolomic data set respectively.

**Fig 2:**
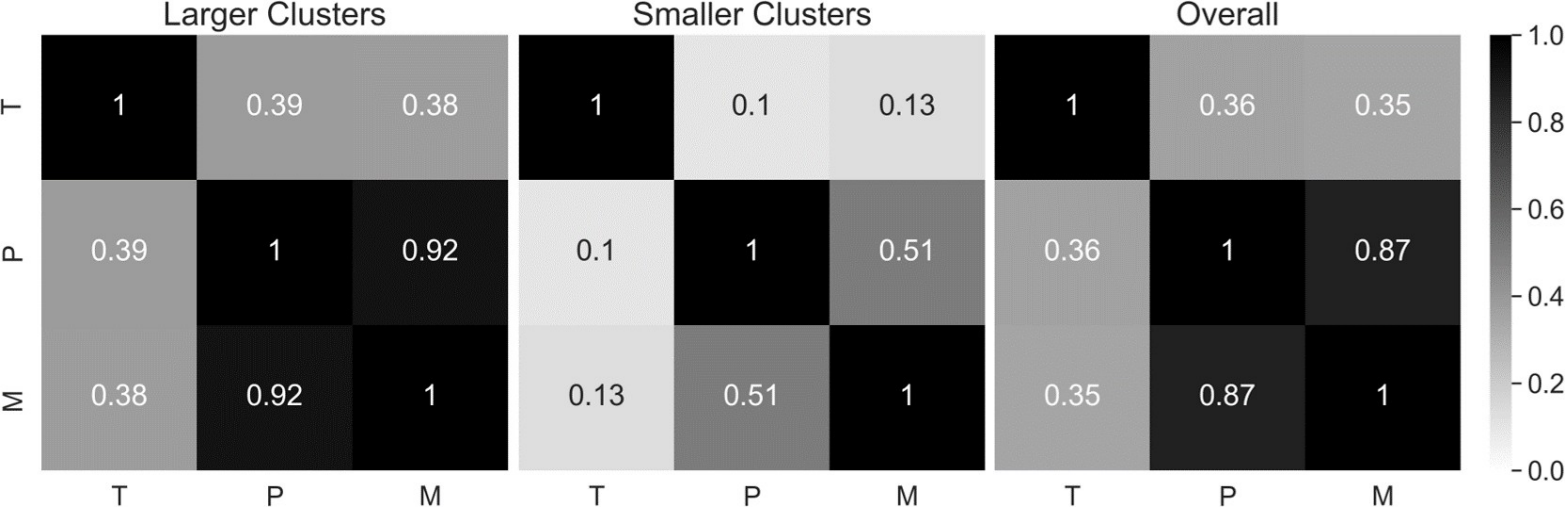
Normalized Jaccard similarity between the clusterings of different datasets. T, P, and M, represent the Transcriptomic, Proteomic or Metabolomic results respectively. The normalized Jaccard accounts for the varying sample sizes for each subtype.

Therefore, we moved forward with AE for dimension reduction, MineClus for clustering, and *k* = 2 (**Table 2**). For the MineClus results, there tended to be one larger subtype (85–90% of subjects), and one smaller subtype (10–15% of subjects) (**S2 Fig in S1 File**). For our final results, we examined the overlap of subjects between the large subtype and found that the metabolomic-proteomic results had the most overlap based on larger values of the normalized Jaccard score (**Fig 2**). Because the second subtype was much smaller, the overlap was not as strong for any pair of -omics data sets, compared to the large clusters. See **S1 Appendix** and **S3–S5 Figs in S1 File** for the sensitivity analysis.

**Table 2:** Final subtyping results based on AE and MineClus for each -omics data. For each -omics type the total number of samples and features, two subtypes (size; silhouette), and number of outliers is listed along with the overall silhouette and connectedness.

Dataset	Samples	Features	w	Outliers	Subtype 1	Subtype 2	Silhouette	Connectedness
Transcriptomics	2637	1889	14.2	23	2342; 0.31	272; 0.35	0.31	0.96
Proteomics	1013	142	5.58	57	848; 0.17	108; 0.13	0.16	0.92
Metabolomics	1057	187	6.5	28	893; 0.20	136; 0.15	0.19	0.92

#### Clinical associations

For each -omics data set, we evaluated the differences on clinical variables between the two subtypes (**Table 3, S5 Table**). Although sometimes there were common discriminating clinical features (e.g., age at enrollment), the subtypes discovered by each of the -omics varied and identified a distinct profile of subjects. The subjects clustering on transcriptomic data differed based on variables such as oxygen therapy, airway wall thickness, and red blood counts. The subjects clustering on proteomic data differed on variables related to kidney disease, blood pressure and smoking duration. The subjects clustering on metabolomics data differed on blood pressure, coronary artery disease, kidney disease, diabetes, and diffusing capacity for carbon monoxide (DLCO). Along with the higher Jaccard scores between the clusters described above, the proteomic-metabolomic results had more overlapping types of clinical variables discriminating the subtypes, e.g., coronary artery disease, kidney disease and several quality of life measurements by the 36-Item Short-Form (SF-36) patient-reported health survey.

**Table 3:** Summary of top 10 single-omics clinical associations. All clinical variables listed were significant at a false discovery rate of 10% over the variables tested and are ordered by significance. Only the top 10 associations are displayed. For more details see Data Dictionary in **S3 Table** and complete results in **S5 Table**.

Transcriptomics	Proteomics	Metabolomics
1-min post-walk Sa02 (%)	Kidney Disease	Age at current visit
Age at current visit	Distance walked (ft)	High Blood Pressure
Airway Wall Thickness, segmental (main 6)	High Blood Pressure	Distance walked (ft)
Red Blood Cell Count	Age at current visit	Coronary Artery Disease
Clinical Center	Duration of smoking (yrs)	Kidney Disease
Heart Rate 1-minute post-walk (beats/min)	Pack years, from Resp Questionnaire	SF-36 Physical Health Aggregate (PCS) Score (normalized)
Hematocrit (%)	Coronary Artery Disease	SF-36 Physical Function (PF) score
Hemoglobin (g/dL)	SF-36 Physical Health Aggregate (PCS) Score (normalized)	SF-36 Physical Function (PF) t-score (normalized)
Resting SaO2 (%)	SF-36 Physical Function (PF) score	Diabetes
In last 12 months, had wheezing or whistling in chest	SF-36 Physical Function (PF) t-score (normalized)	SF-36 Role Physical (RP) t-score (normalized)

#### Feature selection

SVMRFE and cross-validation identified the top 13 transcripts, 41 proteins and 12 metabolites that discriminated between the subtypes (**Table 4, S6 Table, S6 Fig in S1 File**). Most of the genes represented by the transcripts and proteins in **Table 4** are connected based on co-expression, co-localization and genetic interactions (**S7 Fig in S1 File**). Since these subsets were too small to perform an enrichment analysis, we relaxed this list to find the top ranking 250, 42, and 28 features respectively (see Methods), and used the original non-covariate filtered set as our background (**Table 5, S7 Table**). For the transcriptome-based subtypes, for the transcripts that discriminated between the subtypes, we found enrichment in 12 gene ontology (GO) categories related to immune response, ribosomal function, and enzymatic activities. While many of these GO categories are quite broad, the immune and response to bacterium categories might reflect associations with age or airway wall thickness. For the proteome-based subtypes, we found no enrichment in any categories for the proteins that discriminated between the subtypes. For the metabolome-based subtypes, we found enrichment in one group “sphingomyelin” for the metabolites that discriminated between the subtypes. This association is encouraging because sphingomyelins are associated with emphysema [15] and the metabolomics clusters were associated with emphysema markers such as DLco, although not emphysema per se.

**Table 4:** Features selected by SVMRFE for each omic type after 5-fold cross-validation. Cumulative score is the classification metric (f1-score) of an SVM used to predict the cluster labels using only the features at and above that feature (e.g., score for Fibroblast growth factor 20 is the result of an SVM trained on feature set [Interleukin-23, Fibroblast growth factor 20]). These scores are not used to select the size of the feature sets because the size is selected before features are ranked (see Methods).

Dataset	Name	Cumulative f1-score
Transcriptomics	SLCO4C1	91.81%
	TNFRSF10B	92.23%
	SNX4	91.81%
	RLF	91.85%
	SELENOW	91.62%
	TPD52L2	92.77%
	PPP1R10	91.47%
	CD80	92.16%
	SNRPB2	92.27%
	RSL24D1	92.04%
	RPL26L1	95.10%
	RPS27L	91.70%
	FOLR2	91.81%
Proteomics	Interleukin-23	89.75%
	Fibroblast growth factor 20	92.47%
	Stromelysin-1	92.36%
	Macrophage-capping protein	92.05%
	C5a anaphylatoxin	92.68%
	Coagulation Factor X	92.47%
	Gelsolin	92.78%
	Trefoil factor 3	92.57%
	Limbic system-associated membrane protein	93.51%
	Mannose-binding protein C	93.51%
	Adhesion G protein-coupled receptor E2	92.57%
	Neural cell adhesion molecule 1, 120 kDa isoform	92.78%
	Apolipoprotein A-I	92.47%
	Follicle stimulating hormone	93.30%
	Glucose-6-phosphate isomerase	92.68%
	A disintegrin and metalloproteinase with thrombospondin motifs 5	93.62%
	Interleukin-1 receptor-like 1	93.10%
	Nidogen-1	93.31%
	72 kDa type IV collagenase	93.10%
	Transforming growth factor-beta-induced protein ig-h3	93.10%
	C-X-C motif chemokine 10	92.99%
	Hemojuvelin	92.99%
	Complement factor B	92.99%
	Bone morphogenetic protein 1	93.20%
	UNANNOTATED (SOMAmer: 9191–8_3)	91.74%
	C-reactive protein	92.89%
	Insulin-like growth factor-binding protein 6	92.78%
	Apolipoprotein B	92.57%
	C-X-C motif chemokine 16	92.15%
	UNANNOTATED (SOMAmer: 5451–1_3)	92.15%
	Tumor necrosis factor receptor superfamily member 10D	92.47%
	UNANNOTATED (SOMAmer: 5349–69_3)	91.74%
	UNANNOTATED (SOMAmer: 8459–10_3)	91.74%
	UNANNOTATED (SOMAmer: 8464–31_3)	91.74%
	SPARC-related modular calcium-binding protein 1	91.32%
	Mast/stem cell growth factor receptor Kit	92.05%
	Ephrin-B1	91.32%
	NT-3 growth factor receptor	92.36%
	UNANNOTATED (SOMAmer: 5115–31_3)	92.36%
	60 kDa heat shock protein, mitochondrial	92.68%
	UNANNOTATED (SOMAmer: 5509–7_3)	91.94%
Metabolomics	dehydroisoandrosterone sulfate (DHEA-S)	91.93%
	3-(3-amino-3-carboxypropyl)uridine*	91.64%
	X– 12117	91.84%
	stearoyl sphingomyelin (d18:1/18:0)	91.84%
	hydroxy-CMPF*	91.45%
	N6-carbamoylthreonyladenosine	92.52%
	N-formylmethionine	92.23%
	sphingomyelin (d18:1/20:1, d18:2/20:0)*	91.55%
	sphingomyelin (d18:1/17:0, d17:1/18:0, d19:1/16:0)	91.84%
	1-palmitoyl-2-linoleoyl-GPC (16:0/18:2)	92.81%
	3-carboxy-4-methyl-5-propyl-2-furanpropanoate (CMPF)	92.32%
	pyroglutamine*	91.84%
Integrated (Transcriptomics + Proteomics + Metabolomics)	UQCRB	74.28%

**Table 5:** Top enrichment pathways from multiple annotation sources. For annotations with a defined hierarchy (gene ontology: GO), p-values were adjusted per-level of the hierarchy (FDR < 0.10). Levels begin from 1, the lowest level of the hierarchy, and increase to the top level, number of levels vary by annotation database.

Dataset	Annotation	Level	Name	FDR (Level)
Transcriptomics	GO Biological Process	4	regulation of immune response (GO:0050776)	3.97 X 10^−2^
		3	membrane invagination (GO:0010324)	4.54 X 10^−2^
		3	response to bacterium (GO:0009617)	6.29 X 10^−2^
		3	positive regulation of immune response (GO:0050778)	8.10 X 10^−2^
		3	positive regulation of cysteine-type endopeptidase activity (GO:2001056)	8.10 X 10^−2^
		5	positive regulation of catalytic activity (GO:0043085)	8.40 X 10^−2^
	GO Cellular Component	5	immunoglobulin complex (GO:0019814)	5.01 X 10^−6^
		4	immunoglobulin complex, circulating (GO:0042571)	1.13 X 10^−2^
		2	ribosome (GO:0005840)	2.13 X 10^−2^
		2	ribosomal subunit (GO:0044391)	2.13 X 10^−2^
		5	extracellular space (GO:0005615)	5.24 X 10^−2^
		1	cytosolic ribosome (GO:0022626)	8.20 X 10^−2^
Proteomics	No Significant Pathways			
Metabolomics	Sub Class*	N/A	Sphingomyelins	3.19 X 10^−2^

* Sub classes for metabolomic features were annotated by Metabolon, Inc.

### Multi-omics analyses

**Fig 3:**
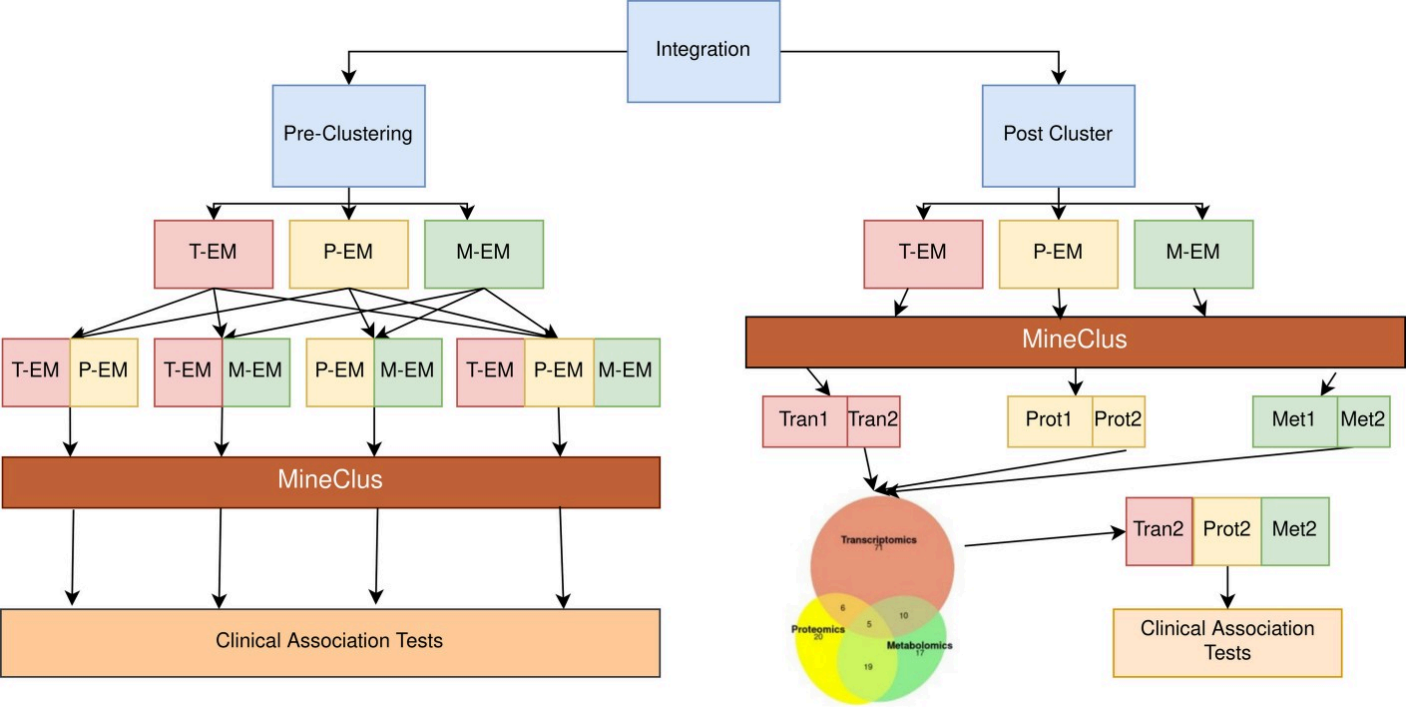
Flow chart for multi-omics pre- and post-clustering integration. T, P, and M, represent the Transcriptomic, Proteomic or Metabolomic data sets respectively. Tran, Prot, and Met represent the Transcriptomic, Proteomic or Metabolomic subtypes respectively. 1 and 2 signify the large or small subtype respectively.

Integration of multi-omics data sets for subtyping can be performed at different steps (**Fig 3**), and we evaluated the effect of integrating either pre- or post-clustering (Steps 3–4, **Fig 1**).

#### Pre-clustering integration

By concatenating the selected embeddings for all of the -omics data sets and then clustering, the maximum silhouette was for AE and MineClus, so we only explored this result. We found two subtypes (k = 2) for each pairwise combination and the combination of all 3 -omic types (**S8 Table**). For the pair-wise omics analyses, the clusters of n = 489 subjects based on transcriptomic and proteomic data differed by clinical center, chest wheezing in the past year, and smoking status. The clusters of n = 511 subjects based on transcriptomic and metabolomic data differed by clinical center and age of enrollment in the study. Finally, the greatest overlap between -omic data types was found for subjects with proteomic and metabolomic profiling, consistent with our previous observations. The clusters of these subjects differed on several variables with the most significant variables being age at enrollment, blood pressure, reported coronary artery disease, distance walked, reported kidney disease, sex, and smoking pack-years. Of note is that the smaller cluster for these subjects had an exceptionally low silhouette (0.04). For the integrated analysis with all three -omics data sets (n = 489), no clinical variables were found to significantly differ between the two subtypes.

#### Post-clustering integration

**Fig 4:**
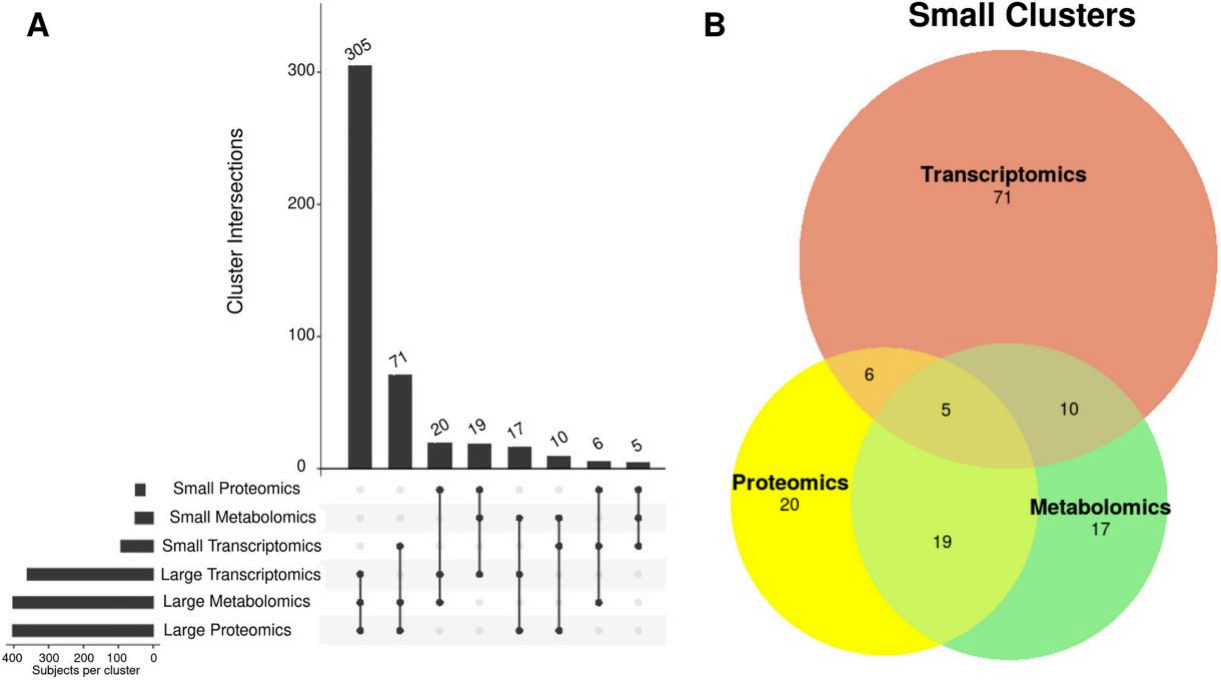
Summary of post-clustering -omics integration. A) Upset plot representing the intersecting clusterings from the single-omic analysis among subjects with all 3 -omic profiles. B) A Venn diagram of the smaller clusters for comparison among subjects specifically categorized in the smaller cluster in only 1 of the 3 -omic clusterings.

We examined the single -omic subtype assignments of subjects with all 3 -omic profiles (n = 489), which resulted in 8 new subtypes based on combinations of whether they were in the large or small subtype (e.g., large transcriptomic, large proteomic, and large metabolomic) (**Fig 4A; S9 Table**) The majority of subjects (77%) were assigned to the large cluster in all -omic subtypings. This new large cluster was not differentiated from the remaining subjects by any clinical variable (**S10 Table**). In exploring the small cluster assignments, there were several subjects assigned to the larger cluster for two of the -omic data types, yet the smaller cluster in the remaining -omic data type (**Fig 4B**). These subjects were of interest based on their distinct profiling within one particular -omic data set. These groups (highlighted in **S9 Table**) were found to be significantly different based on clinical measures, potentially identifying new subtypes (**Table 6**). The subtype based on the smaller transcriptomic cluster identified younger and healthier subjects than the metabolomic and proteomic profiles, as evident with significantly longer average distance-walked, higher diffusing capacity of the lungs for carbon monoxide (DLCO), less gas trapping, and increased FEV_1_ percent predicted compared to the other 2 groups. In contrast, the subtype based on the smaller metabolomic cluster were older and more diseased than the other two groups. Finally, the subtype based on the smaller proteomic cluster had the lowest average 6-minute walk distance of the three groups, and percent on atrial fibrillation. Otherwise, that subtype was in between the two other groups with respect to age and lung function.

**Table 6:** Post-clustering clinical associations of subjects that are in the small subtype for one of the -omics data sets, but not the others.

Clinical variable	FDR*	Small Transcriptomic, Large Proteomic & Metabolomic	Small Proteome Cluster, Large Transcriptomic & Metabolomic	Small Metabolome, Large Transcriptomic & Proteomic
Distance walked	4.91E-02	1397.3 (308.4)	1020.0 (520.2)	1184.4 (584.6)
DLco percent predicted	5.44E-02	95.2 (25.3)	75.5 (23.8)	69.5 (27.1)
Atrial Fibrillation ^a^	5.44E-02	5.6%	0.0%	35.3%
Percent gas trapping (-856)	6.80E-02	14.5 (15.0)	26.2 (22.1)	30.4 (19.9)
Age at enrollment	6.80E-02	65.2 (8.3)	70.7 (7.3)	73.4 (10.3)
FEV1 percent predicted	7.41E-02	83.2 (21.7)	69.5 (28.4),	61.6 (18.0)

*FDR adjusted p values over the variables tested. For more detailed explanation of the clinical variables see Data Dictionary in **S3 Table**. Mean and standard deviation except for ^a^ which is reported as a percentage.

#### Stratified analysis

To assess whether subsets of COPDGene subjects had different profiles, we performed the clustering only on subjects stratified by COPD cases status, or current/former smokers (**S11 Table**). In all cases, we compared how the clinical associations varied in the stratified results compared to the set of all subjects. For COPD cases, subtyping by each of the individual -omics data sets did not identify subtypes that differentiated by any clinical variables for proteomics, and only age enrolled for metabolomics, and oxygen saturation for transcriptomics. For smoking status, we identified two subtypes each for current and former smokers separately for each -omics data set. The clinical associations that differentiated the two subtypes changed based on smoking status, especially for the proteomic and transcriptomic profiles. Based on proteomic data, subtypes of current smokers differed mostly by age, duration smoking, and DLCO, while subtypes of former smokers mostly differed by reported kidney disease, platelet counts, diabetes status, age, distance walked, and airway wall thickness. Based on metabolomic data, subtypes of current smokers differed by blood counts, while subtypes of former smokers differed mostly by sex and airway wall thickness.

## Discussion

In this work, we developed a pipeline for the largest multi-omics subtyping of COPD, which complements subtyping approaches using clinical or imaging data [6, 8, 11]. We found that when the subtypes of each -omics profile were explored separately, clustering by omics was complementary and each omics platform tended to identify unique clusters of subjects with different clinical features. Although there was some overlap, especially between the metabolomics and proteomic results, there were also unique clinical signatures associated with subtypes for each of the three -omics subtypes. In particular, prominent molecular features of the metabolomics clustering included sphingomyelin (which has known associations with emphysema), while key transcriptome pathways were immune and bacterial responses. Similarly, some key associations with the proteomic molecular clusters include kidney disease, for which the SOMAscan platform has been particularly useful for clustering [16]. These findings suggest that comorbidities such as diabetes, metabolic syndrome, and renal failure, may play a strong role in subtyping and need to be considered in multi-omic clustering.

We explored existing methods for -omics integration which are broadly categorized as horizontal and vertical integration methods [17]. Horizontal methods integrate subjects from different datasets of the same -omic type (e.g., multiple RNA-Seq datasets), which are not applicable to our analysis. Of the remaining vertical integration methods, they can be further categorized as parallel or hierarchical. Hierarchical methods focus the analysis on one -omics data at a time and in sequence (e.g., from gene to protein level) while preserving information of the previous analysis in the next which requires some form of annotation to associate -omic features across datasets. Of the parallel methods, mixOmics [18] is the most similar system to the one used in this study, and provides a number of methods to integrate -omics, including PCA, PLS, and CCA. However, none of these methods were able to provide the same level of internal metrics and clinical feature associations as integration using concatenated embeddings.

Our study generated important insights on whether integration should be done pre- or post-clustering for the COPD data. For instance, pre-clustering (concatenating all three profiles and treating them equally) did not identify consistent subtypes that differed on clinical variables. In the post-clustering approach (examining overlapping subjects from subtypes based on each profile separately), we found that subjects that were not consistently assigned to the large subtype for all -omics data sets differentiated themselves based on certain clinical associations. For example, the subtype based on the smaller metabolomic cluster were older and more diseased than the other combinations. Post-clustering approaches that integrate across clustering results based on individual data types have been used in several cancer applications (e.g., [19, 20]).

For observational studies of human diseases, covariates such as sex and age can be highly associated with the disease signal. But these covariates can also be strongly associated with molecular profiles in the blood. As a first pass, we included all clinical features but found that the results from the clustering algorithms primarily separated male versus females, current versus former smokers, or by cell types, as there are strong gene, protein or metabolic signatures for these differences. Although these results could be useful for future investigations, we wanted to focus on clusters that were more specific to COPD disease severity and progression. However, if we only include features that separate based on COPD severity (e.g., GOLD stage) this resorts to a supervised learning approach based on existing classifications, making it more difficult to identify new subtypes. Therefore, we found covariate filtering in Step 1 and removal of basic (but influential) covariates such as sex and age to be an important step that allowed us to focus on disease-related profiles rather than demographic variables. We did this in an unbiased manner by testing 242 available clinical variables.

Although the covariate filtering step helped reduce dimensionality, there were still hundreds or thousands of features depending on the -omics data set which can be problematic for clustering algorithms. Dimension reduction for more than two omics datasets is essential computationally. High dimensional datasets are hard to visualize, and complete enumeration of all subspaces becomes computationally intractable with increasing dimensionality [21]. We identified AE as a particularly useful dimension reduction strategy that preserved a lot of the original variance. We also used other methods for dimension reduction such as PCA, and partial-least squares regression and report the former. The subtyping results on PCA versus AE were very similar based on the clustering metrics, but the AE embeddings more closely recapitulated the original data based on mean squared error. For both PCA and AE, we used cross-validation and “elbow-plots” to determine the number of dimensions for clustering.

For clustering, we evaluated other standard methods such as K-means, and hierarchical clustering, and report the former. However, given a large number of attributes, it is likely that some attributes are correlated. Hence, clusters might exist in arbitrarily oriented dense but isolated subspaces, therefore we focused on subspace clustering, as implemented in MineClus [22]. Among all methods explored, subspace clustering algorithms performed the best as they do not require inclusion of potentially irrelevant features in clustering; hence, more realistic clusterings. In our application, K-means did not perform as well as subspace clustering. It may not be ideal given the large number of remaining but essential data dimensions, even after significantly reducing the dimensionality of the dataset.

The subtyping results are highly dependent on the MineClus tuning parameter *w*, and number of clusters *k*, so we used several resampling approaches, and systematic testing of *w* to make the final selection. We found that the results were stable with respect to *w*. However, we found that often some values of *w* resulted in two subtypes where the smaller one had a much smaller silhouette score. To ensure that both subtypes were cohesive, we required a minimum silhouette score (>0.10) for the smaller subtype when possible. The best number of subtypes was typically *k =* 2 based on multiple clustering metrics. However, the gap statistic analysis indicated that the proteomic data showed less evidence of clustering as *k =* 1 was preferred.

There are some important limitations to this work. First, the overlap of the proteomic-metabolomic results throughout our analyses may be due to more similar characteristics and recruitment centers of subjects with these data, compared to subjects with transcriptomic data. However, the similarities between the proteomic-metabolomic results also held when we constrained the analyses to overlapping subjects with all three data sets. In the future, through programs such as TOPMed, more subjects will be profiled with these -omics technologies which will increase the overlap of subjects between the datasets. Second, because COPDGene is currently the only study focused on COPD with -omics data in relatively large numbers, there is no appropriate replication sample for the analyses performed. Third, although manageable with eight dimensions, MineClus was not scalable when the dimensions were greater than 30. Our cross-validation process for selecting the number of dimensions did not indicate values that high, but this may not be the case for other studies. Finally, subtyping can be very unstable in some cases. Since most of the subspace clustering methods resort to randomization to address the combinatorial complexity of evaluating clusters in numerous possible subspaces, these methods can be unstable depending on tuning parameters and also sensitive to outliers [23]. Therefore, at every step in our workflow, we evaluated the stability of our results by trying different methods, evaluating different metrics, applying cross-validation strategies, and performing sensitivity analyses.

## Conclusion

Our work illustrates the benefits of subtyping based on multi-dimensional molecular profiles, and the importance of using multiple profiles for providing different perspectives on disease severity. We recommend exploring each single -omics separately first, followed by a multi-omics approach. For -omics integration, one of the critical decisions is when to perform the integration, i.e., early or late in the process [24]. There are benefits and advantages for each approach, and the best solution often depends on the specific hypothesis and data set (see reviews on integration in [25, 26]). In our work, we noticed that the early integration did not result in subtypes that had meaningful clinical differences. This may be reflecting one of the drawbacks of early integration, which disregards the unique distribution of each of the -omics data types and treats them equally [24]. For this problem and data set, we found that focusing on subjects that clustered differently amongst the -omics data sets highlighted more interesting clinical characteristics.

## Materials and methods

### Data

#### Study population and ethics statement

The NIH sponsored multicenter Genetic Epidemiology of COPD (COPDGene, ClinicalTrials.gov Identifier: NCT00608764) study was approved and reviewed by the institutional review board at all participating centers [27]. All study participants provided written informed consent (**S1, S2 Tables** for demographics clinical centers, and IRB boards for each center). For information on recruitment dates, inclusion/exclusion criteria for participant recruitment, and descriptions of where participants were recruited, please see Regan et al., 2010 [27]. Data analyzed were de-identified. Each in-person visit included spirometry before and after albuterol, quantitative computed tomography (CT) imaging of the chest, and blood sampling. In this work, we focus on -omics data sets collected at Phase 2 based on blood sampling (**Table 1**), which have the most extensive contemporaneous -omics datasets.

#### Clinical data and definitions

Of the 808, clinical variables available on subjects [27], we selected 273 that directly measured COPD phenotypes (see **S3 Table**). The remaining variables could be grouped roughly into demographic variables (e.g., sex, age), clinical blood count measures, medical history, symptom questionnaires, and clinical imaging. See **S1 Appendix** for case definitions.

#### Transcriptomics

Transcript profiles were quantified for 2,650 subjects (1980 Non-Hispanic Whites—NHW, 667 African Americans—AA) from 20 clinical centers in the COPDGene Phase 2 study (**Table 1, S1, S2 Tables, S1 Appendix**) from total RNA extracted from whole blood samples.

#### Proteomics

Proteomic profiles were generated on 1,013 subjects (933 NHW; 80 AA) from two clinical centers (National Jewish Health and University of Iowa) who participated in an ancillary study in which they provided fresh frozen plasma collected using an 8.5 ml p100 tube (Becton Dickinson) at Phase 2 (**Table 1, S1, S2 Tables, S1 Appendix**). The proteomic data was quantified at National Jewish Health using the SOMAscan^Ⓡ^ Human Plasma 1.3K assay.

#### Metabolomics

Metabolite profiles from p100 samples from Phase 2 were generated on 1,136 subjects (1,040 NHW; 96 AA) using the Metabolon (Durham, USA) Global Metabolomics Platform, as described [28, 29] (**Table 1, S1, S2 Tables, S1 Appendix**).

### Omics data adjustment, scaling and covariate filtering

All datasets, having previously been log transformed, were adjusted for blood cell counts by regressing measures for hemoglobin, neutrophil percent, lymphocyte percent, eosinophil percent, monocyte percent and whole blood count against the molecular profiles, and extracting the residuals from the regression fit. These residuals were then centered and scaled using Z-score standardization. Next, features were filtered based on significant associations with at least one of 242 clinical or demographic variables (having removed blood count and demographic variables from the aforementioned 273) associated with lung function, COPD, or other comorbidities (**S3 Table, S1 Appendix**). To avoid bias due to outliers, non-parametric tests were used to identify association: either a Wilcoxon signed rank or Kruskal-Wallis based on whether the variable was continuous or categorical respectively. We accounted for multiple testing using the false discovery rate (FDR) procedure of Benjamini and Hochberg [30]. Features remained if they were significantly associated with at least one clinical variable at a stringent threshold (within variable FDR adjusted p value < 10^−5^) to help optimize the efficiency of the dimension reduction step.

### Principal components analysis (PCA) implementation

The implementation of PCA used in this study is from the scikit-learn package in Python 3. The number of principal components (PCs) was chosen by first transforming the dataset to the largest number of allowable PCs (minimum of either the sample size *n*, or number of features *p*), then plotting the percent variance explained for each component.

### Autoencoder (AE) implementation

A deep autoencoder network (hereafter simply called autoencoder) is a genre of deep neural networks for identifying a reduced representation of a given dataset. This representation differs from other methods such as PCA or Independent Component Analysis in that it is non-linear. Autoencoders consist of an encoder and decoder pair of multi-layer neural networks such that the encoder network learns an embedding of each data point into a latent space (typically with fewer dimensions than the input space) that the decoder network then learns to revert back into the original input space (**Fig 1**). Training of an autoencoder involves estimating the latent space that captures the information of the data points in the input space in a reduced form so that it may be decoded efficiently. In this case, efficiency is measured in terms of mean squared error between a decoded data point and the corresponding input data point. A test set (randomly chosen 10% subset of the total subjects) is held out to measure the error on unseen data for each epoch and to check for overfitting of the model, which we define as a >5% difference in the train/test error. Once training is completed, only the encoder network is needed to reduce the dimensionality of the input dataset.

### Dimension reduction

We used the “elbow” method to find the number of dimensions where the gains in silhouette (defined below) or mean square error for AE or percent variance explained for PC, leveled off (see **S1 Appendix**). The same number of dimensions was used for all -omics datasets to facilitate the subsequent multi-omics analysis and avoid data set dominance.

### Clustering

In order to select the clustering algorithm for this study, we examined various methods, including K-Means [31], sparse K-Means [32], spectral clustering [33], hierarchical clustering [34], and subspace clustering [35]. For all algorithms, to decide the number of clusters (*k*), we tested each dataset with different clustering algorithms, and explored clusterings with 2–10 clusters. We found that, based on the various evaluation metrics discussed below, *k* in the range of [2–4] performed the best. In this work, we focus on results obtained from K-means as well as MineClus, which is an implementation of subspace clustering (see **S1 Appendix**).

### Metrics

Different internal and external cluster quality metrics were used to select the best clustering algorithm and the best number of clusters (*k*) and *w*. These metrics were also used to assess the quality of the clusterings and are described in the **S1 Appendix**. Briefly, the silhouette coefficient measures the quality of a clustering [36] based on the cohesion and separation of its clusters, and the connectedness score [37] evaluates the compactness of the clusterings.

### Sensitivity analysis

Because clustering algorithms can be sensitive to parameters, we examined the sensitivity of our clusters and cluster size to perturbations using resampling based methods described in more detail in the **S1 Appendix**. Briefly, we used the gap statistic [38], which is an alternative for the elbow method that is widely used to determine the optimal number of clusters *k*, and the Jaccard similarity, which is commonly used to measure the similarity between two sets A and B. In addition, we examined the stability of clusters, and the consistency of membership of subjects in clusters.

### Clinical associations

Subjects were assessed for cluster assignment associations with the aforementioned 273 clinical variables using an ANOVA for continuous variables, a chi-squared test for categorical variables with expected frequencies greater than 5, and a Fisher’s exact test if the expected frequencies were less than 5. We considered associations statistically significant at p-value < 0.05 after employing a FDR correction for multiple testing [30].

### Feature selection

Feature selection was implemented using Recursive Feature Selection based on Support Vector Machines (SVMRFE) [39]. SVMRFE works by iteratively removing one or more features from the feature set at a time based on how well each feature performs in discerning the outcome variable (here the cluster labels). Performance of a feature in this case is the square of its corresponding coefficient from a linear SVM trained on that feature set. The features are therefore ranked by the reverse order of the iteration in which each is removed (*e*.*g*., the feature removed in the iteration before the last is ranked second). The optimal number of features is chosen as the feature set that optimizes the accuracy of the SVM in predicting the outcome variable and all such features are ranked first. We visualized connections between the top features in a network framework using GeneMANIA [40].

### Enrichment

The number of features used in enrichment differed from the selected features in order to have a good representation of pathways. Because feature set sizes can be ranked by the classification metric (e.g., f1-score), if too few features were selected to generate meaningful enrichment results (e.g., <20), more features are chosen for the analysis by moving further down the rankings to include more features (see **S1 Appendix**). We took all features with an f1-score above 0.93, a percentage point above the average score of 0.92. The analysis was then performed on each of the datasets using Fisher’s Exact Test with FDR adjusted p-values using the top features as defined above ranked from the feature selection process.

### Integration

We explored integration of the -omics data at two different stages of the pipeline. First, we integrated pre-clustering by clustering on the concatenated autoencoder embeddings from each of the -omics types. In this method for integration, each of the -omics datasets were embedded using a separate autoencoder unique to each -omic datasets, then joined by subject identifiers. For the purposes of integration, we required that each -omic type was represented by the same length of embeddings (e.g., proteomics and metabolomics would both have embeddings of length 8, making 16 features in the integrated dataset). The number of embeddings were determined using the methods described above. This restriction on embedding length prevented any one -omic type from dominating the clusters, and thus, the clinical associations and selected features. We explored the same dimension reduction and clustering methods described above. Second, we integrated post-clustering, where subjects with all three omic profiles were reclassified based on their single -omic cluster assignments. Clinical associations were assessed among the subjects uniquely identified in the smaller cluster for an individual -omic type.

### Stratified analyses

We further explored clusterings, clinical associations, and selected features within each -omic profile. Specifically, we stratified by COPD case status (to explore further clusterings within individuals), and current smoking status (to avoid confounding based on smoking). We clustered using the methods described above.

## Supporting information

S1 Table Recruitment centers of subjects profiled by each of the three -omics technologies. (DOCX)

S2 Table Clinical characteristics and demographics for subjects profiled by all -omics technologies. (DOCX)

S3 Table Data dictionary of clinical variables. “Variable name” and “Label” are abbreviations and descriptions for each variable respectively, and “Form” denotes the questionnaire filled out by the clinical coordinator to obtain that variable. The “Use” column indicates which variables were used, either during the filtering step (“Filter”) or clinical association testing (“Test”).
(XLSX)

S4 Table Exploration of subtyping methods. Results are displayed for different algorithms (k-means; KM or MineClus: MC), dimension reduction (autoencoder; AE, or principal components; PC), values of k and w (MC only), along with the number of outliers (MC only). For each subtype the size and silhouette are listed, along with the overall silhouette and connectedness. Final results are highlighted in yellow.
(DOCX)

S5 Table Clinical variables associated with the two subtypes in each -omics data. Columns include clinical variable, type of statistical test, p-value, FDR and summary statistics (mean/percent) for this clinical variable for each subtype. For more detailed explanation of the clinical variables see Data Dictionary in **S3 Table**.
(XLSX)

S6 Table Ranks for all features as determined by recursive feature elimination using SVM (SVMRFE). (XLSX)

S7 Table Complete enrichment results. Tables include all levels of annotation hierarchy for gene ontology (GO). Sorted by FDR calculated for each level of the hierarchy if applicable. Ontologies or pathways less than FDR < 0.01 reported in the manuscript are highlighted in red, in addition to genes in these categories.
(XLSX)

S8 Table Summary of pre-clustering -omics integration. (DOCX)

S9 Table Post-clustering subtype assignments of subjects based on single -omic data. (DOCX)

S10 Table Post-clustering clinical associations of subjects in large subtype for all three -omics data sets compared to all other subjects. Columns include clinical variable, type of statistical test, p-value, FDR and summary statistics (mean/percent) for this clinical variable for each subtype. For more detailed explanation of the clinical variables see Data Dictionary in **S3 Table.**
(XLSX)

S11 Table Results from stratified analysis compared to the full data analysis. AE, MineClus and k = 2 were used for each -omics data. For each subtype the size and silhouette are listed, along with the number of outliers, top clinical associations, and overall silhouette and connectedness.
(XLSX)

S1 File Supporting figures. (DOCX)

S1 Appendix Supporting methods and results. (DOCX)
