# Supplementary material for: Multi-omics subtyping pipeline for chronic obstructive pulmonary disease
Source: PLoS One. 2021 Aug 25;16(8):e0255337. doi: 10.1371/journal.pone.0255337 (PMC8386883; doi:10.1371/journal.pone.0255337)
Supplement: S1 Table — (DOCX) [file pone.0255337.s001.docx]

**S1 Table: Recruitment centers of subjects profiled by each of the three -omics technologies**

| Center* | Transcriptomic | Proteomic | Metabolomic |
| --- | --- | --- | --- |
| % NJC | 13.6 | 63.6 | 63.7 |
| % UIA | 15.4 | 36.4 | 36.3 |
| % AVA | 0 | 0 | 0 |
| % BAY | 2.1 | 0 | 0 |
| % BWH | 4.8 | 0 | 0 |
| % COL | 6.7 | 0 | 0 |
| % DUK | 1.4 | 0 | 0 |
| % FAL | 5.4 | 0 | 0 |
| % HAR | 6.8 | 0 | 0 |
| % HPR | 3.7 | 0 | 0 |
| % JHU | 5 | 0 | 0 |
| % MSM | 3.3 | 0 | 0 |
| % MVA | 0.8 | 0 | 0 |
| % PIT | 9 | 0 | 0 |
| % TEM | 7.7 | 0 | 0 |
| % TXS | 2.4 | 0 | 0 |
| % UAB | 3.9 | 0 | 0 |
| % UMC | 3.6 | 0 | 0 |
| % UMN | 0.4 | 0 | 0 |
| % USD | 4.2 | 0 | 0 |
| AVA=Ann Arbor VA,BAY=Baylor,BWH=Brigham,COL=Columbia,DUK=Duke,FAL=Reliant Medical Group,HAR=Harbor-UCLA,HPR=Minnesota HealthPartners,HVA=HoustonVA,JHU=Johns Hopkins,MSM=Morehouse School of Medicine,MVA=Minneapolis VA,NJC=National Jewish,PIT=Pittsburgh,TEM=Temple,TXS=U of Texas,UAB=U of Alabama,UIA=U of Iowa,UMC=U of Michigan,UMN=U of Minnesota,USD=UC San Diego | | | |
