# Supplementary material for: Multi-omics subtyping pipeline for chronic obstructive pulmonary disease
Source: PLoS One. 2021 Aug 25;16(8):e0255337. doi: 10.1371/journal.pone.0255337 (PMC8386883; doi:10.1371/journal.pone.0255337)
Supplement: S2 Table — (DOCX) [file pone.0255337.s002.docx]

**S2 Table: Clinical characteristics and demographics for subjects profiled by all -omics technologies.**

| **Variable** | **Overlap** |
| --- | --- |
| No. of participants | 489 |
| Age (mean(sd)) | 67.5 (8.5) |
| % Female | 49.9 |
| % Male | 50.1 |
| %AA | 8.4 |
| % NHW | 91.6 |
| BMI (mean(sd)) | 29.2 (6.3) |
| % Former Smoker | 76.7 |
| % Current Smoker | 23.3 |
| Pack Years | 45.3 (25) |
| % Controls | 52.6 |
| % COPD cases | 47.4 |
| % PRISm | 10.9 |
| % GOLD 0 | 41.6 |
| % GOLD 1 | 10.7 |
| % GOLD 2 | 20.4 |
| % GOLD 3 | 10.7 |
| % GOLD 4 | 5.6 |
| FEV_1_pp (mean(sd)) | 77.2 (25.6) |
| FEV_1_/FVC (mean(sd)) | 0.7 (0.2) |
| % Emphysema (mean(sd)) | 7.3 (10.7) |
| Exacerbation Frequency (mean(sd)) | 0.2 (0.7) |
| % Chronic Bronchitis | 16.6 |
| % missing | 29 (5.9) |
| % NJC | 67.3 |
| % UIA | 32.7 |
| sd-standard deviations; COPD is defined by GOLD score > 0; PRISm - Preserved Ratio Impaired Spirometry (18);NHW - Non-Hispanic White,; AA - African American; BMI – body mass index (kg/m2); FEV1/FVC = post-bronchodilator forced expiratory volume at one second (FEV1)/forced vital capacity (FVC); FEV1pp = FEV_1_ percent predicted; Chronic bronchitis was defined as self-reported chronic cough and sputum for at least three months in each of the two years prior to baseline. Quantitative emphysema was quantified by percent of lung voxels -950 Hounsfield Units (% low attenuation areas: %LAA) on the full inspiratory CT scans. Visual emphysema was assessed as described by (2). Exacerbations were defined as acute worsening of respiratory symptoms requiring treatment with oral corticosteroids and/or antibiotics, emergency room visit, or hospital admission (3).The Wilcoxon rank test was used for sex, smoking status, COPD status, and chronic bronchitis status; ANOVA were used for age, bmi, smoking pack-years, FEV1pp, FEV1/FVC, percent emphysema, and exacerbation frequency; chi-square test was used for COPD severity by GOLD & PRISm status. | |
