# Supplementary material for: Multi-omics subtyping pipeline for chronic obstructive pulmonary disease
Source: PLoS One. 2021 Aug 25;16(8):e0255337. doi: 10.1371/journal.pone.0255337 (PMC8386883; doi:10.1371/journal.pone.0255337)
Supplement: S4 Table — Results are displayed for different algorithms (k-means; KM or MineClus: MC), dimension reduction (autoencoder; AE, or principal components; PC), values of k and w (MC only), along with the number of outliers (MC only). For each subtype the size and silhouette are listed, along with the overall silhouette and connectedness. Final results are highlighted in yellow. (DOCX) [file pone.0255337.s004.docx]

**S4 Table: Exploration of subtyping methods** Results are displayed for different algorithms (k-means; KM or MineClus: MC), dimension reduction (autoencoder; AE, or principal components; PC), values of k and w (MC only), along with the number of outliers (MC only). For each subtype the size and silhouette are listed, along with the overall silhouette and connectedness. Final results are highlighted in yellow.

| **Dataset** | **Samples** | **Features** | **Algorithm** | **AE or PC** | **k** | **w** | **Out-**  **liers** | **Sub-type 1** | **Subtype 2** | **Subtype 3** | **Silhouette** | **Connectedness** |
| --- | --- | --- | --- | --- | --- | --- | --- | --- | --- | --- | --- | --- |
| Transcript-omics | 2637 | 1889 | KM | AE | 2 | NA | 0 | 1696; 0.31 | 941; 0.20 | - | 0.27 | 0.93 |
|  |  |  | KM | PC |  | NA | 0 | 1696; 0.33 | 941; 0.23 | - | 0.29 | 0.94 |
|  |  |  | MC | AE |  | **14.2** | **23** | **2342; 0.31** | **272; 0.35** | **-** | **0.31** | **0.96** |
|  |  |  | MC | PC |  | **25** | **264** | **1913; 0.33** | **460; 0.36** | **-** | **0.34** | **0.94** |
|  |  |  | KM | AE | 3 | NA | 0 | 1083; 0.14 | 969; 0.19 | 585; 0.16 | 0.16 | 0.88 |
|  |  |  | KM | PC |  | NA | 0 | 1087; 0.16 | 968; 0.21 | 582; 0.18 | 0.18 | 0.89 |
|  |  |  | MC | AE |  | 11.08 | 201 | 1753; 0.30 | 375; 0.07 | 308; 0.10 | 0.24 | 0.88 |
|  |  |  | MC | PC |  | 28.1 | 77 | 1833; 0.29 | 422; 0.13 | 305; 0.04 | 0.25 | 0.88 |
| Prote-omics | 1013 | 142 | KM | AE | 2 | NA | 0 | 583; 0.21 | 430; 0.05 | - | 0.14 | 0.86 |
|  |  |  | KM | PC |  | NA | 0 | 585; 0.21 | 428; 0.03 | - | 0.13 | 0.85 |
|  |  |  | MC | AE |  | **5.58** | **57** | **848; 0.17** | **108; 0.13** | **-** | **0.16** | **0.92** |
|  |  |  | MC | PC |  | **7.32** | **60** | **849; 0.25** | **104; 0.18** | **-** | **0.24** | **0.92** |
|  |  |  | KM | AE | 3 | NA | 0 | 550; 0.23 | 375; 0.05 | 88; 0.10 | 0.15 | 0.84 |
|  |  |  | KM | PC |  | NA | 0 | 376; 0.20 | 360; 0.07 | 277; 0.10 | 0.13 | 0.81 |
|  |  |  | MC | AE |  | 5.34 | 31 | 653; 0.15 | 215; 0.06 | 114; 0.05 | 0.12 | 0.80 |
|  |  |  | MC | PC |  | 8.12 | 21 | 690; 0.19 | 188; 0.09 | 114; 0.01 | 0.15 | 0.82 |
| Metabol-omics | 1057 | 187 | KM | AE | 2 | NA | 0 | 625; 0.19 | 432; 0.09 | - | 0.15 | 0.86 |
|  |  |  | KM | PC |  | NA | 0 | 732; 0.16 | 325; 0.13 | - | 0.15 | 0.88 |
|  |  |  | MC | AE |  | **6.5** | **28** | **893; 0.20** | **136; 0.15** | **-** | **0.19** | **0.92** |
|  |  |  | MC | PC |  | **8.52** | **98** | **850; 0.20** | **109; 0.18** | **-** | **0.20** | **0.91** |
|  |  |  | KM | AE | 3 | NA | 0 | 410; 0.13 | 373; 0.14 | 274; 0.09 | 0.12 | 0.80 |
|  |  |  | KM | PC |  | NA | 0 | 432; 0.12 | 367; 0.16 | 258; 0.12 | 0.13 | 0.81 |
|  |  |  | MC | AE |  | 3.9 | 189 | 589; 0.13 | 148; 0.09 | 131; 0.01 | 0.10 | 0.79 |
|  |  |  | MC | PC |  | 9.6 | 30 | 708: 0.10 | 199; 0.15 | 120; 0.12 | 0.11 | 0.82 |
