# Supplementary material for: Multi-omics subtyping pipeline for chronic obstructive pulmonary disease
Source: PLoS One. 2021 Aug 25;16(8):e0255337. doi: 10.1371/journal.pone.0255337 (PMC8386883; doi:10.1371/journal.pone.0255337)
Supplement: S8 Table — (DOCX) [file pone.0255337.s008.docx]

**S8 Table: Summary of pre-clustering -omics integration.** MineClus was used with eight AE embeddings and k=2, for all pairwise -omics analysis, and integrative analysis. Top clinical associations are included for each comparison. See **S4 Table** for labels. Red values indicate silhouette values < 0.10.

| **Dataset** | **Samples** | **w** | **Outliers** | **Cluster 1** | **Cluster 2** | **Silhouette** | **Connectedness** | **Clinical Assocations** |
| --- | --- | --- | --- | --- | --- | --- | --- | --- |
| Transcriptomics and Proteomics | 490 | 10.82 | 48 | 322; 0.20; | 120; 0.26 | 0.21 | 0.87 | ccenter, ChestWheez12mo, smoking_status |
| Transcriptomics and Metabolomics | 511 | 10.46 | 43 | 346; 0.18 | 122; 0.26 | 0.20 | 0.88 | ccenter, Age_Enroll |
| Proteomics and Metabolomics | 1008 | 6.52 | 47 | 841; 0.19 | 120; 0.04 | 0.17 | 0.90 | Age_Enroll, HighBloodPres, CoronaryArtery, Diabetes, distwalked, KidneyDisease, gender, AFib, CongestHeartFail, ATS_PackYears, DLCOpp, CABG, lymphcyt, SF36_PCS_score, AWT_seg_Thirona, SF36_RP_t_score, SF36_RP_score, SF36_PF_t_score, SF36_PF_score, diasBP, hemoglobin |
| Transcriptomics and Proteomics and Metabolomics | 489 | 13.68 | 3 | 436; 0.19 | 50; 0.17 | 0.18 | 0.92 | Nothing Significant |
