# Supplementary material for: Multi-omics subtyping pipeline for chronic obstructive pulmonary disease
Source: PLoS One. 2021 Aug 25;16(8):e0255337. doi: 10.1371/journal.pone.0255337 (PMC8386883; doi:10.1371/journal.pone.0255337)
Supplement: S9 Table — (DOCX) [file pone.0255337.s009.docx]

**S9 Table: Post-clustering Subtype Assignments of Subjects Based on Single -Omic Data.**

| Combination of single -omics subtypes | No. of Subjects |
| --- | --- |
| Large Transcriptomic, Large Proteomic, Large Metabolomic | 305 |
| **Small Transcriptomic, Large Proteomic, Large Metabolomic** | **71** |
| **Large Transcriptomic, Large Proteomic, Small Metabolomic** | **17** |
| **Large Transcriptomic, Small Proteomic, Large Metabolomic** | **20** |
| Small Transcriptomic, Large Proteomic, Small Metabolomic | 10 |
| Small Transcriptomic, Small Proteomic, Large Metabolomic | 6 |
| Large Transcriptomic, Small Proteomic, Small Metabolomic | 19 |
| Small Transcriptomic, Small Proteomic, Small Metabolomic | 5 |
| *Clusterings in bold indicate the groups of subjects used for the post-clustering integration analysis. | |
