# Supplementary material for: Multi-omics subtyping pipeline for chronic obstructive pulmonary disease
Source: PLoS One. 2021 Aug 25;16(8):e0255337. doi: 10.1371/journal.pone.0255337 (PMC8386883; doi:10.1371/journal.pone.0255337)

**S1 Figures: Supporting Figures**

**S1a Figure: Dimension reduction for Autoencoder.** Plots of max silhouette (grey) and mean squared error (MSE) for the training (orange) and test set (yellow) for different number of AE output nodes. The MSE is cumulative over the number of nodes, and the silhouette calculated on the clusters resulting from a run of k-means with k=2.


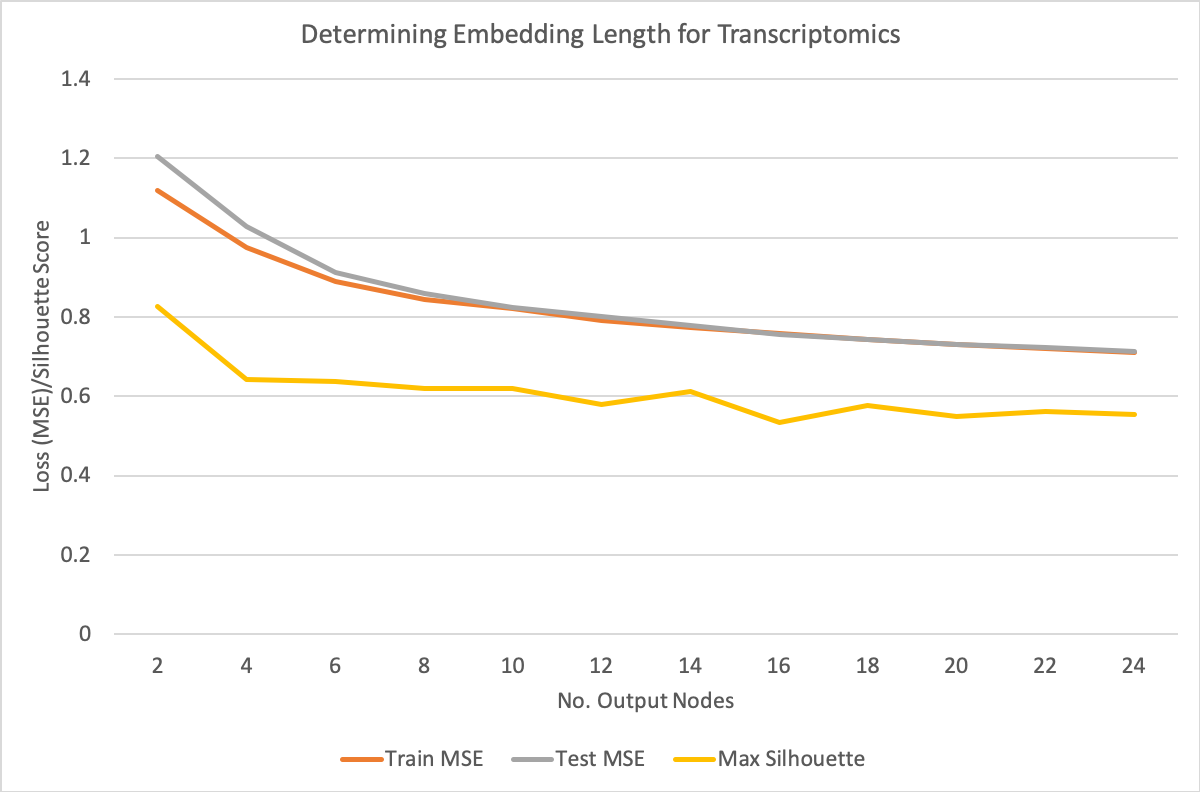


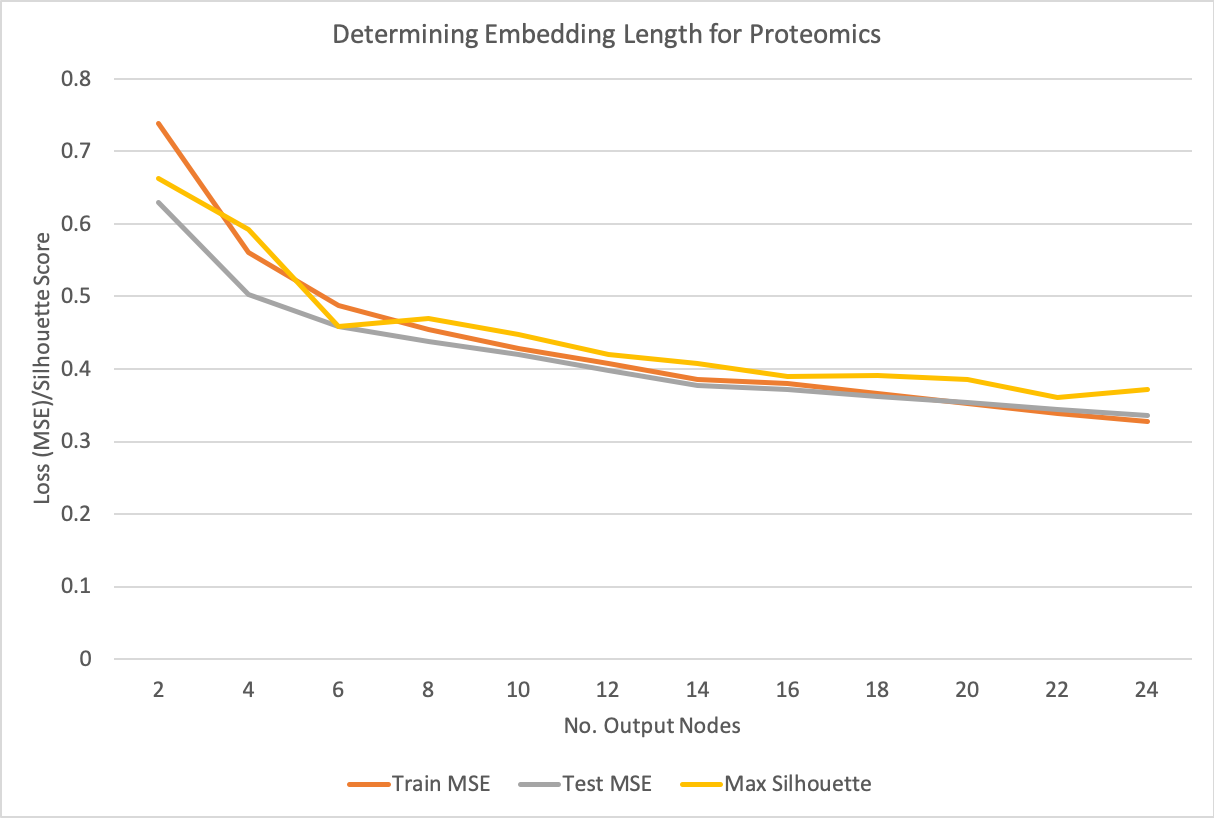

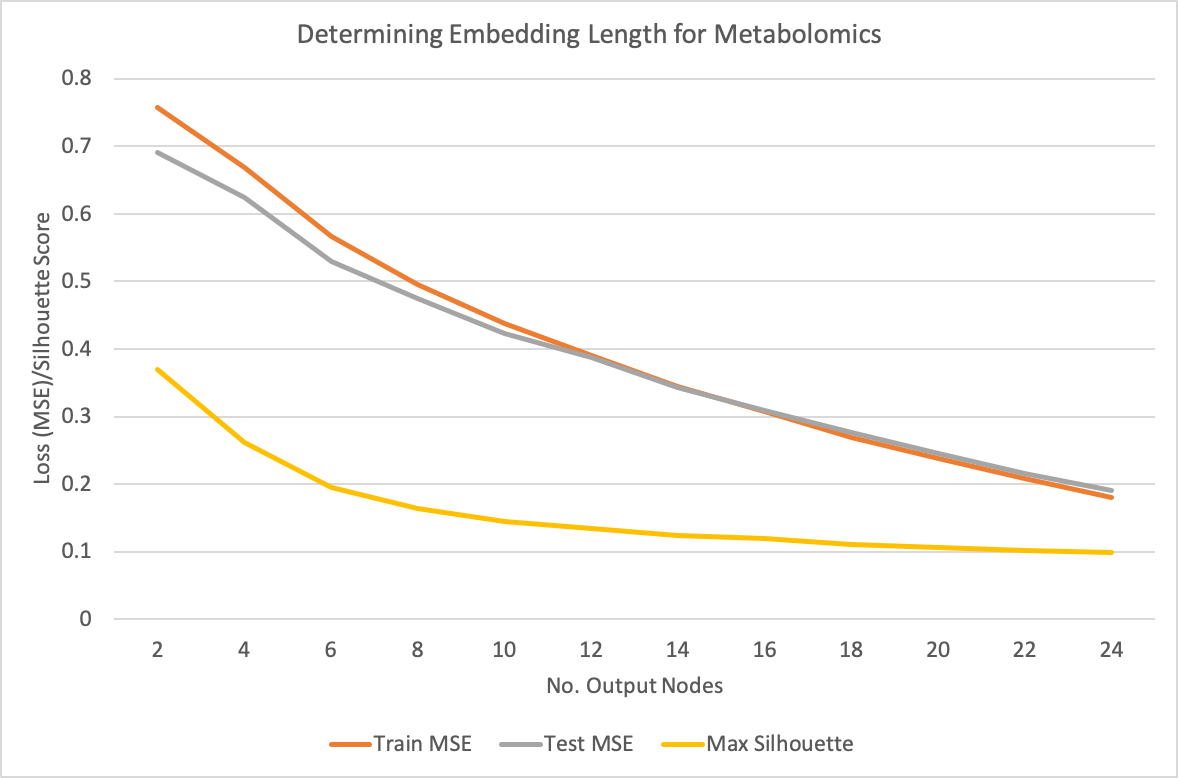


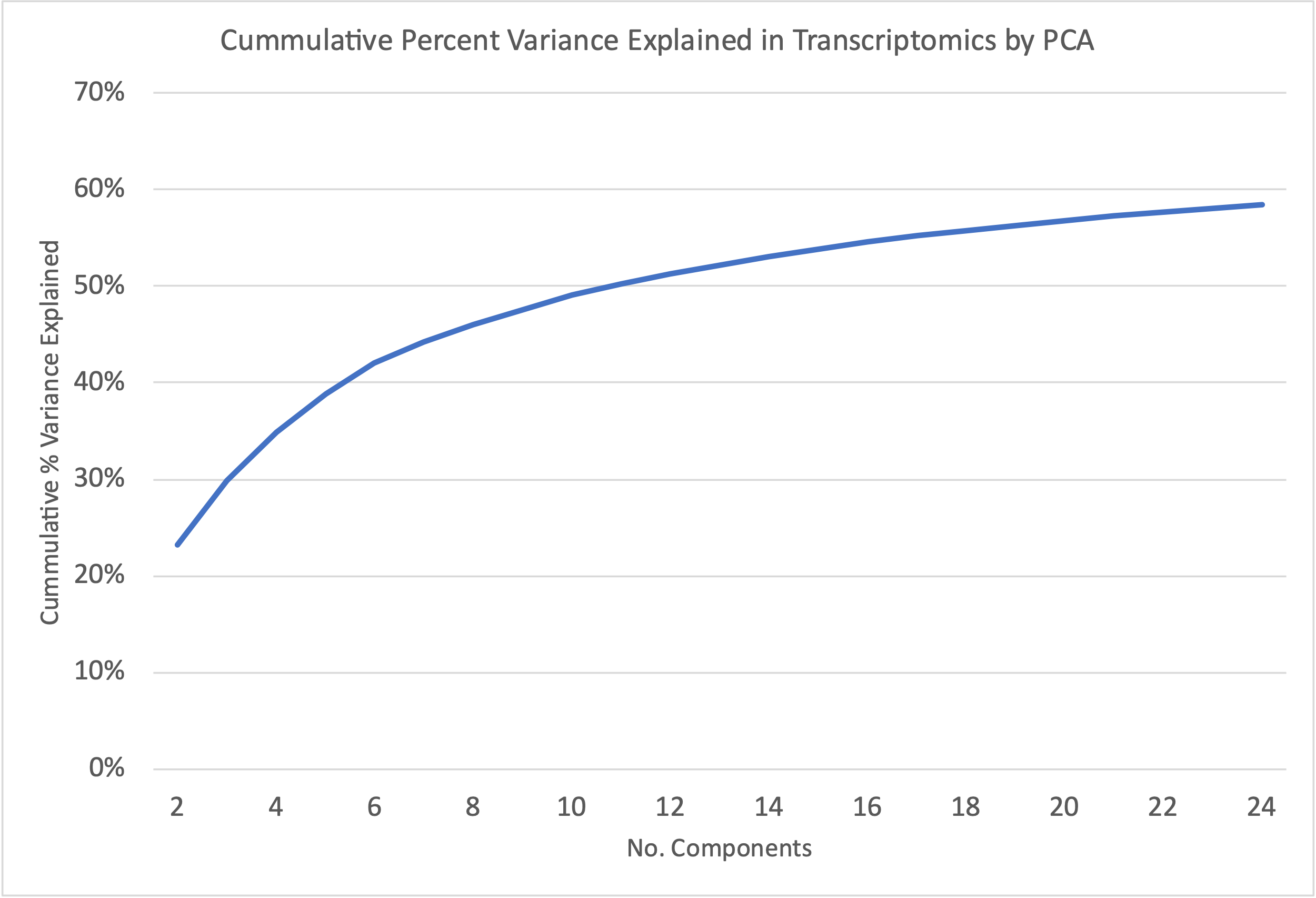

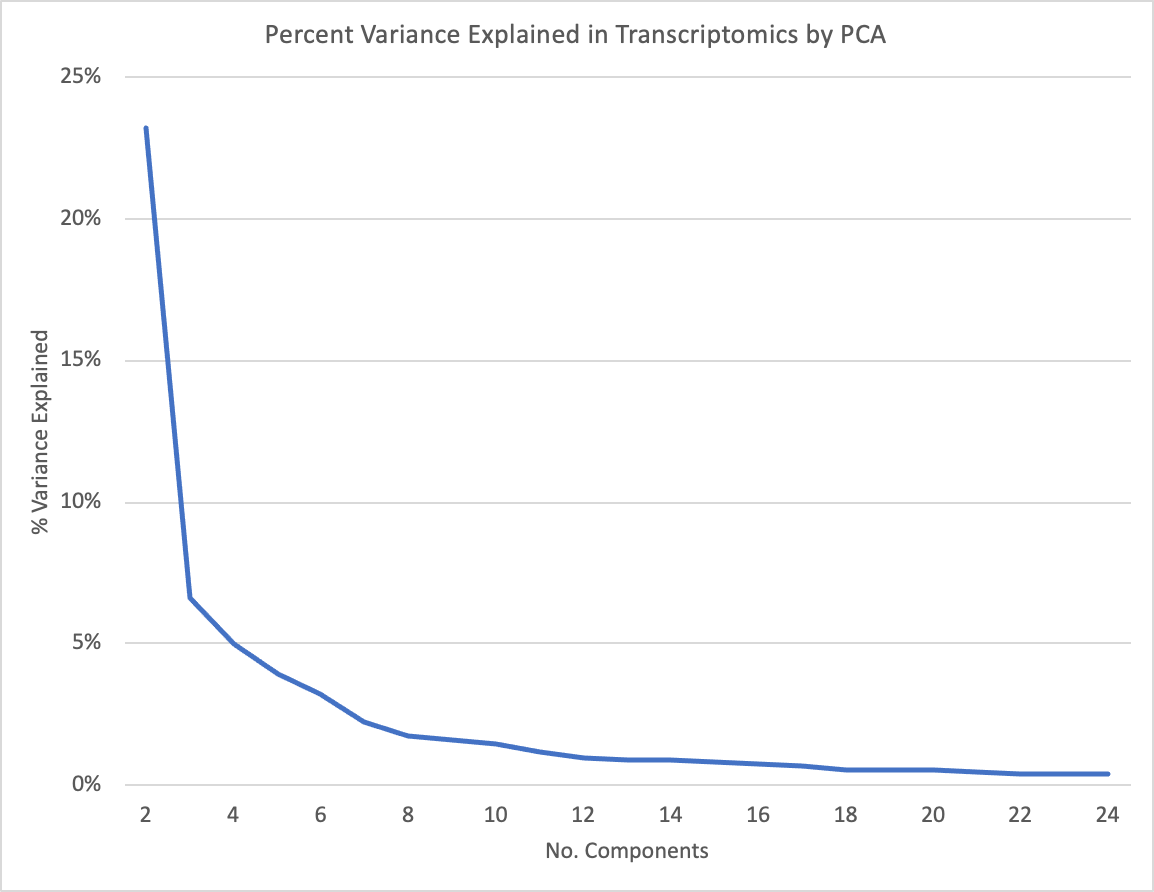
**S1b Figure: Dimension reduction for PCA.** Plots of variance explained by each component individually (left) and cumulatively (right) for different numbers of principal components.


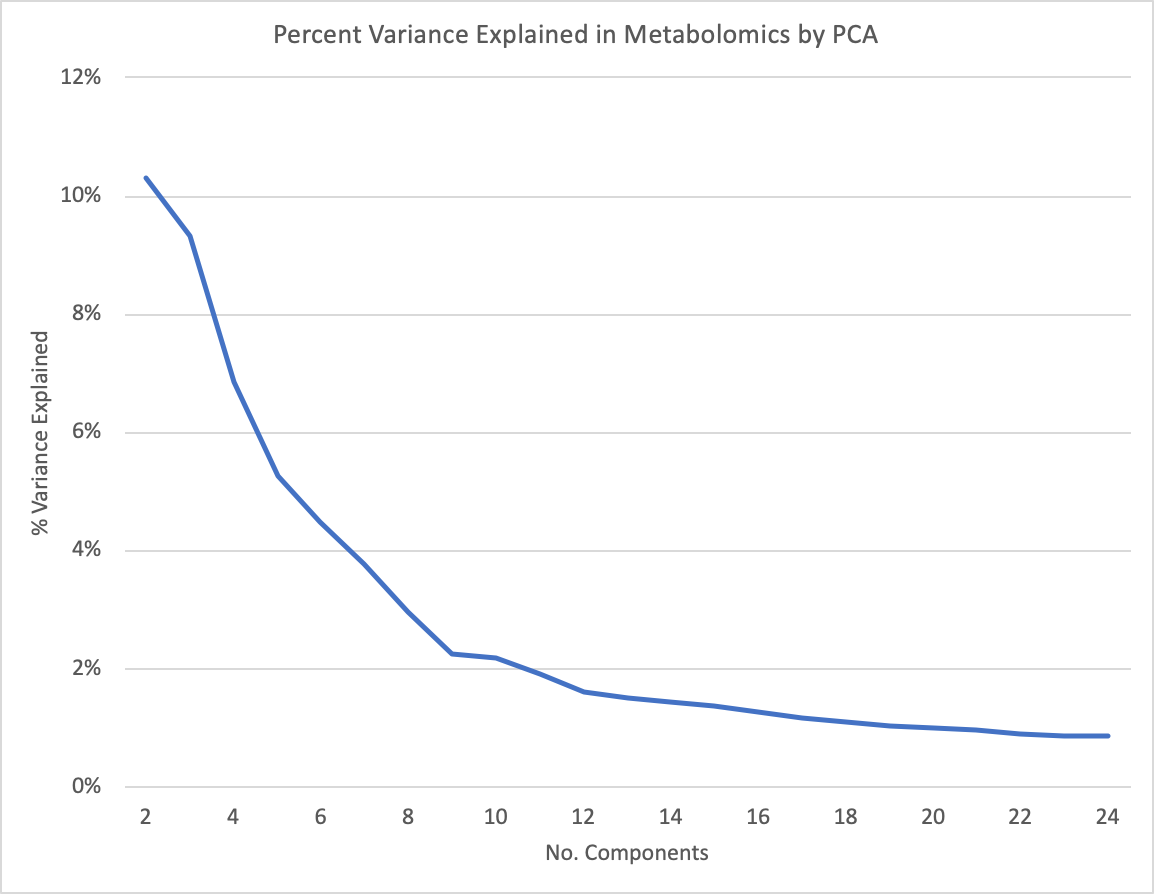

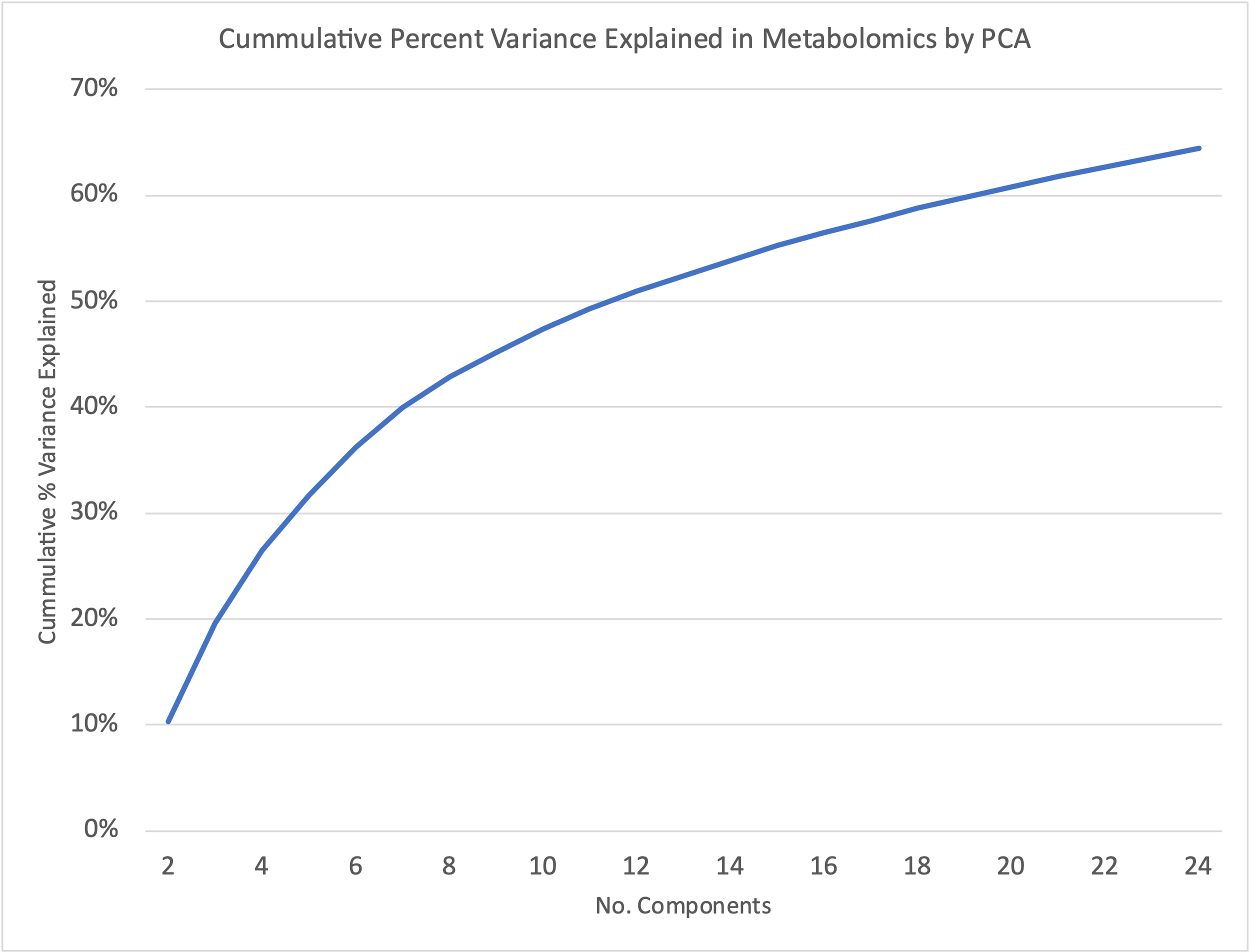

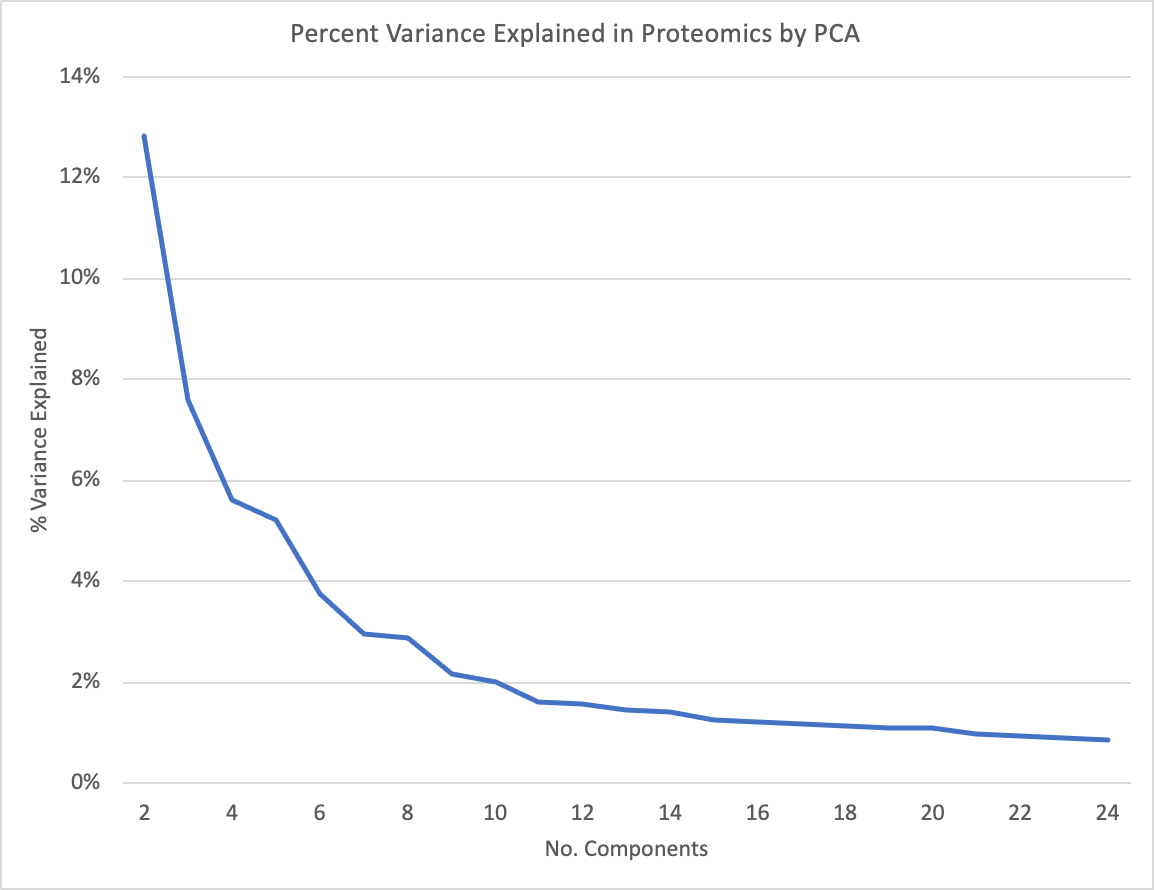

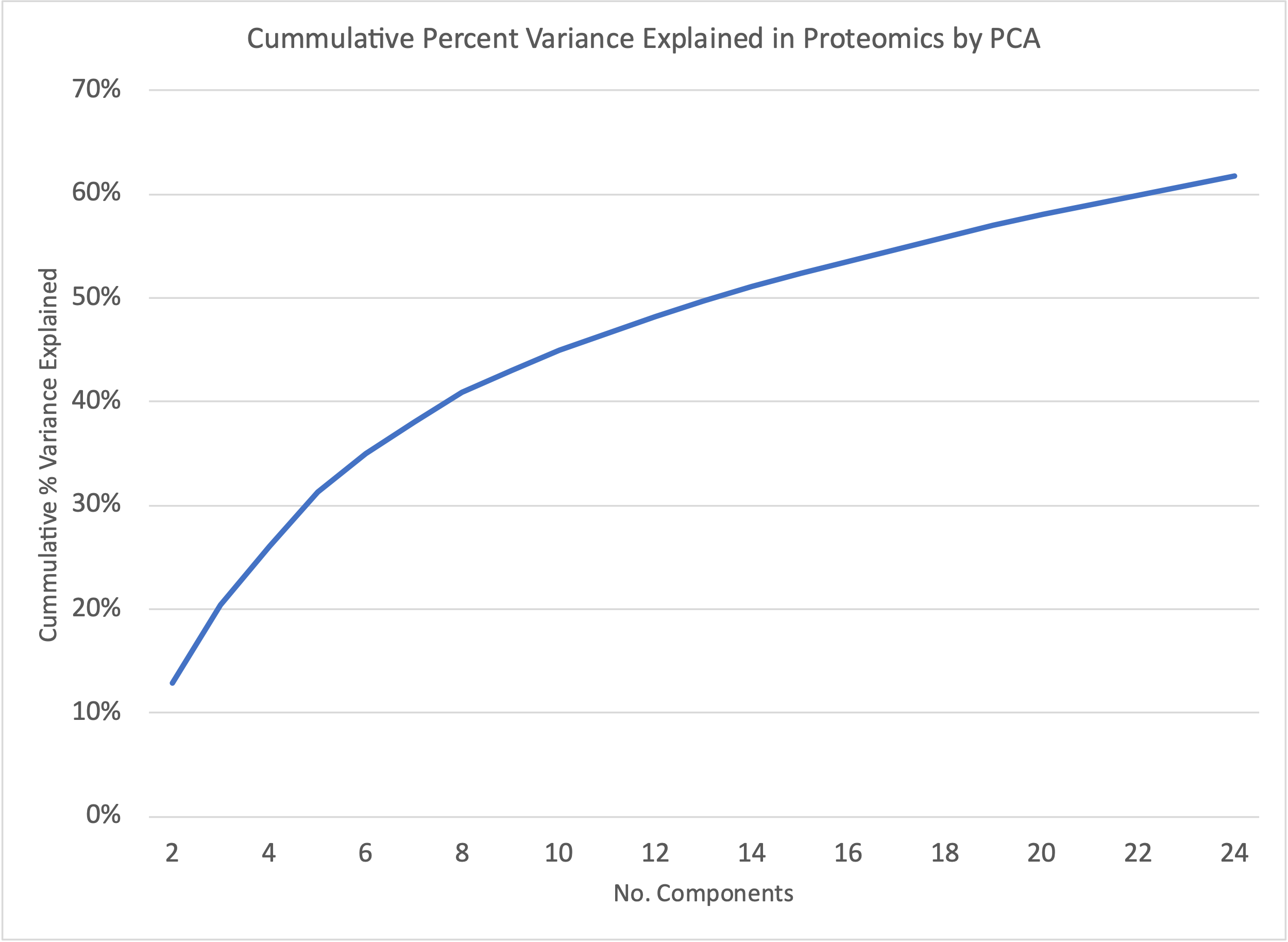


**S2 Figure: Visualization of clusters.** Subjects are colored by final subtype assignment for the first two principal components of the original data.

**
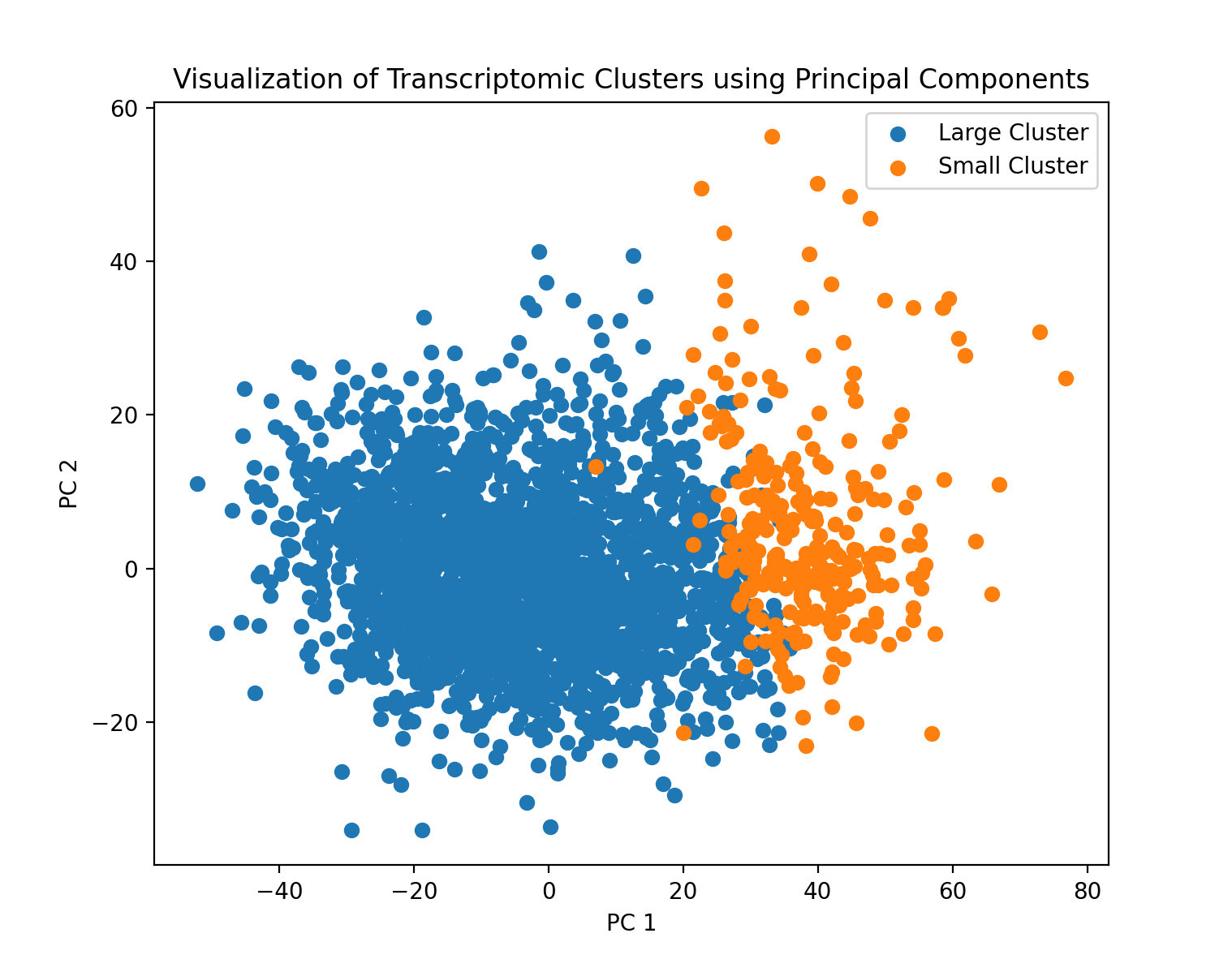

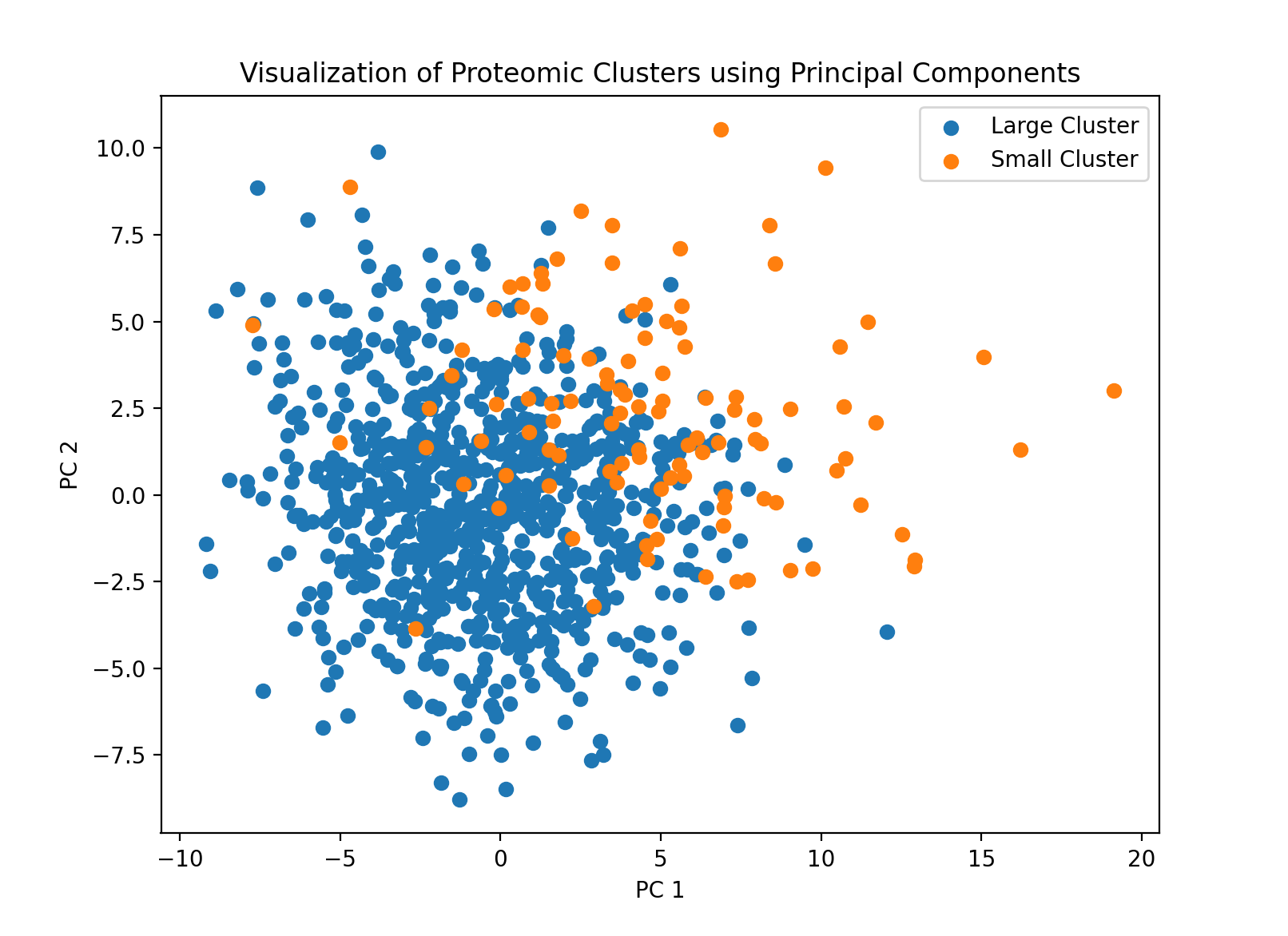
**

**
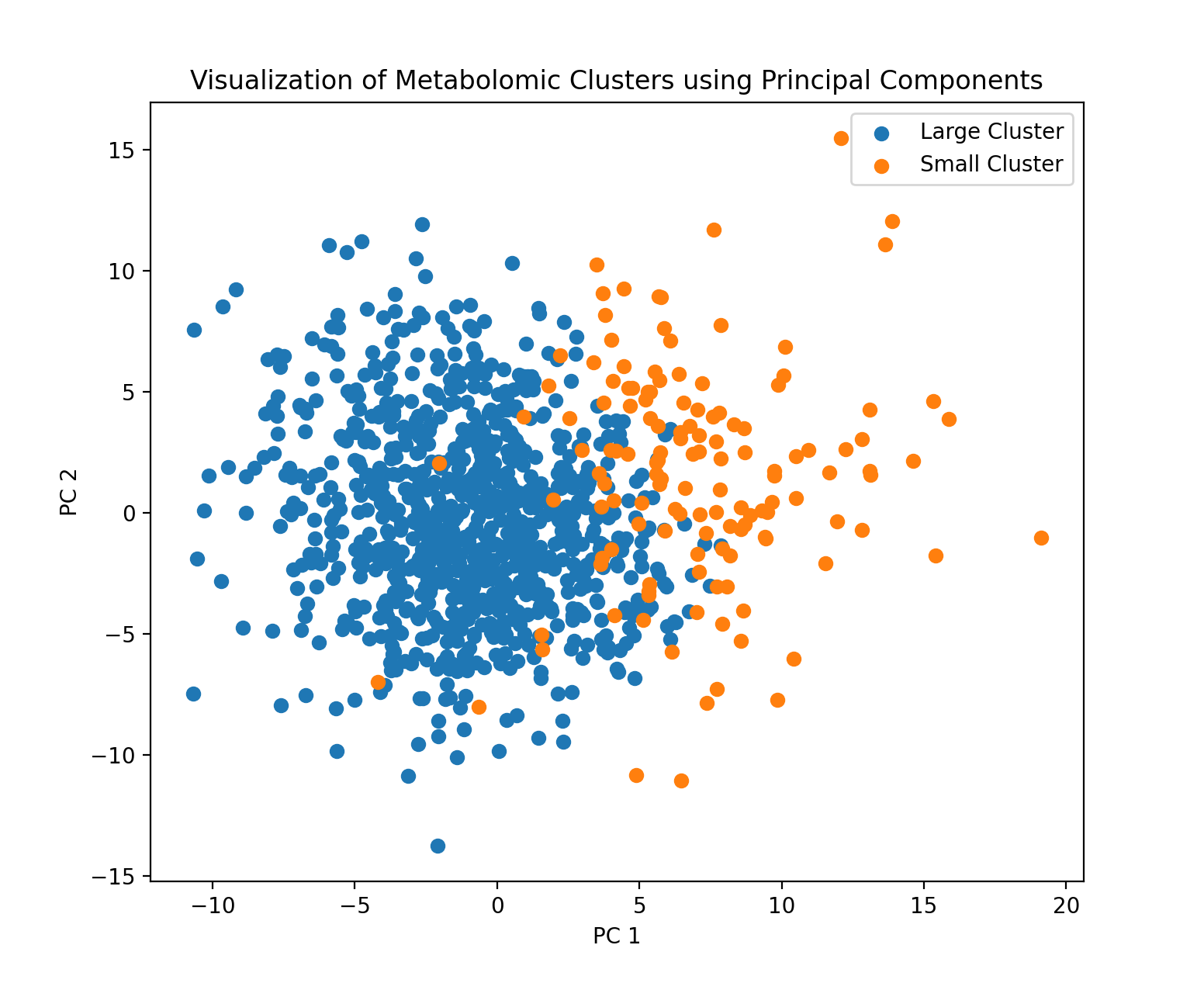
**

**S3 Figure: Gap statistic analysis to explore number of clusters, *k*.**  Error bar indicates the standard deviation of the intra-cluster distances for the 20 normal-random datasets for each *k*. Larger values of the gap statistic indicate that the clustering in the original data set is more compact compared to the clusterings for the random datasets. However, the gap statistic increases as *k* increases. The authors suggested if *gap(k)* is within one standard deviation of *gap(k+1*), k should be adequate, which is achieved at *k*=3 for Transcriptomics, *k*=1 for Proteomics, and *k*=4 for Metabolomics.


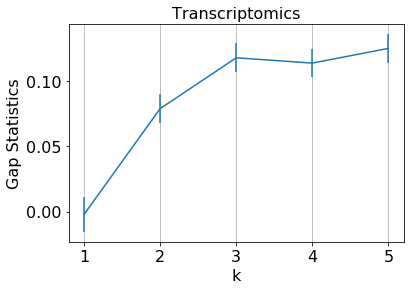

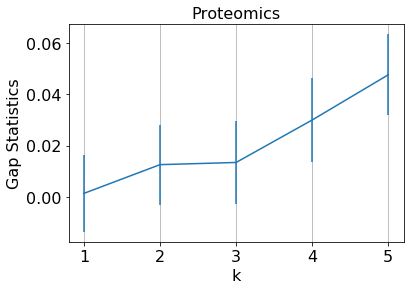


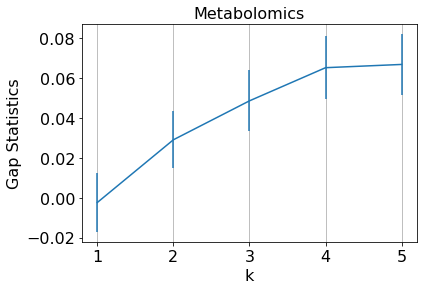


**S4 Figure:** **Sensitivity Plots for MineClus *w* tuning parameter.** The dashed black line shows the *w* with the highest overall silhouette, while the solid black line shows the *w* with the highest silhouette, where the silhouette score of the smaller cluster is at least 0.10. These lines overlap for the Transcriptomics results. Stability refers to the stability metric for clusterings only when *k*=2.


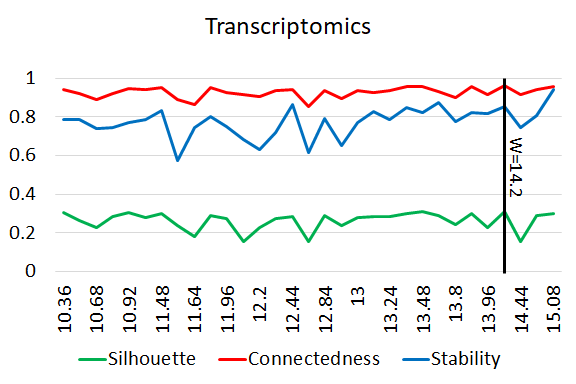

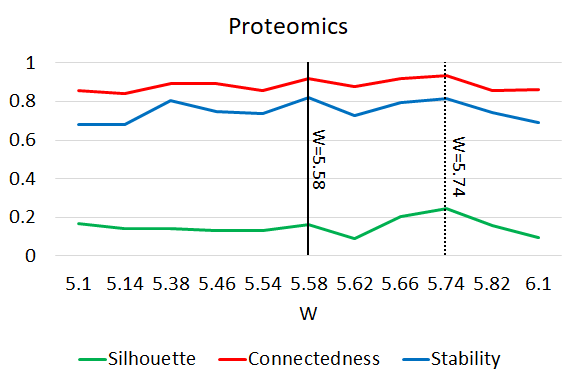

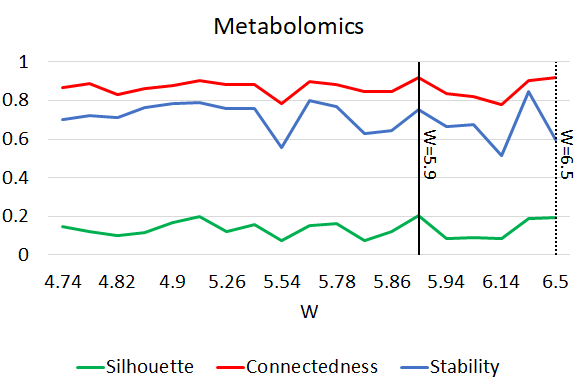


**S5 Figure: Stability of membership of subtypes.** The membership strength of each subject is displayed for each of the 3 possible clusters: larger, smaller, and outliers. For example, if there are 10 clusterings, and Subject #1 was in the outlier/larger/smaller clusters 2/5/3 times, the RGB tuple will be (0.2, 0.5, 0.3), which is mapped to
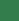
. PC1 and PC2 are the first and second principal components derived from the original data.


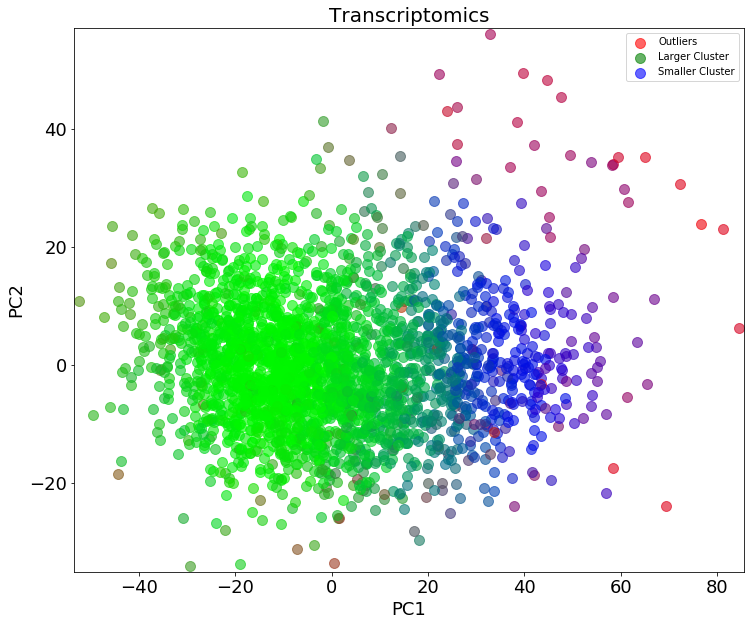

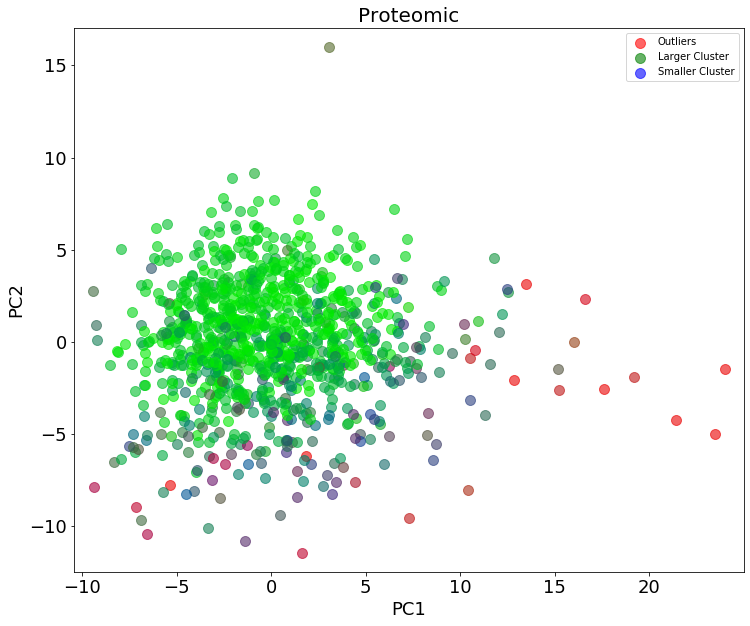

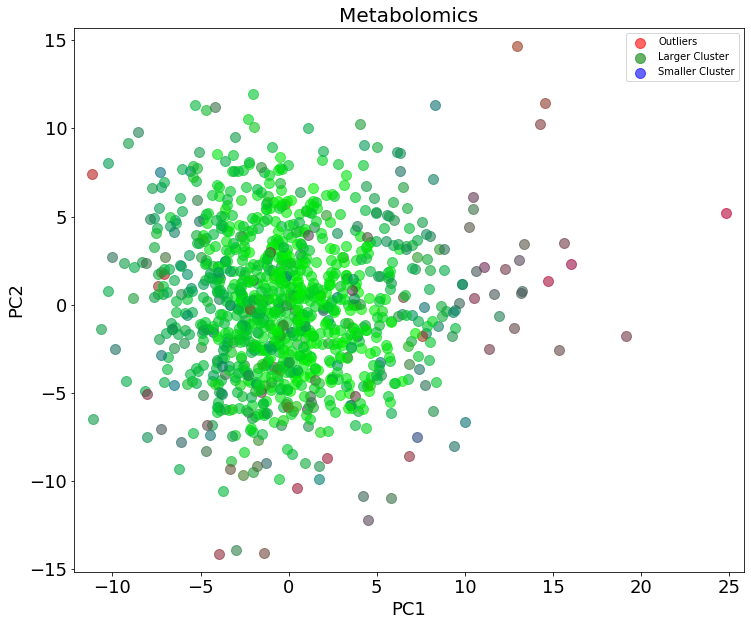


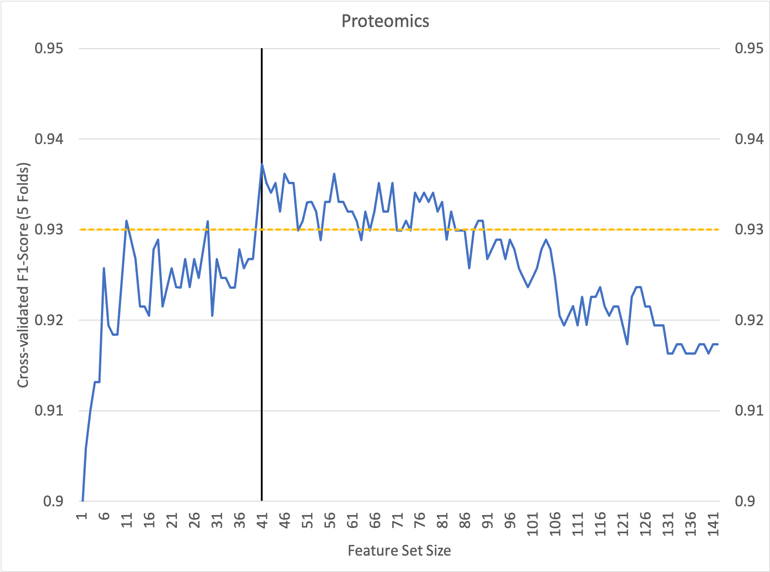

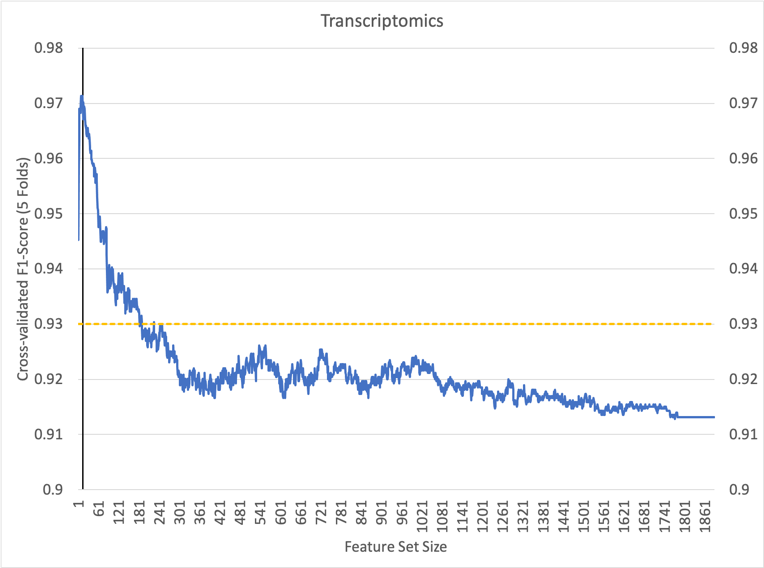
**S6 Figure: Curve of feature set performance used to determine the selected set of features using SVMRFE.** Averaged SVM classification scores over 5-folds of validation. From the full set of features (left) the lowest scoring feature based on SVM weight is removed to form the next point and repeated until a single point remains. The selected feature set corresponds to that which maximizes the curve (achieved at solid black line). The threshold for adding more features to the enrichment analysis is indicated by the orange dotted line (for proteomics only the selected features were used instead).


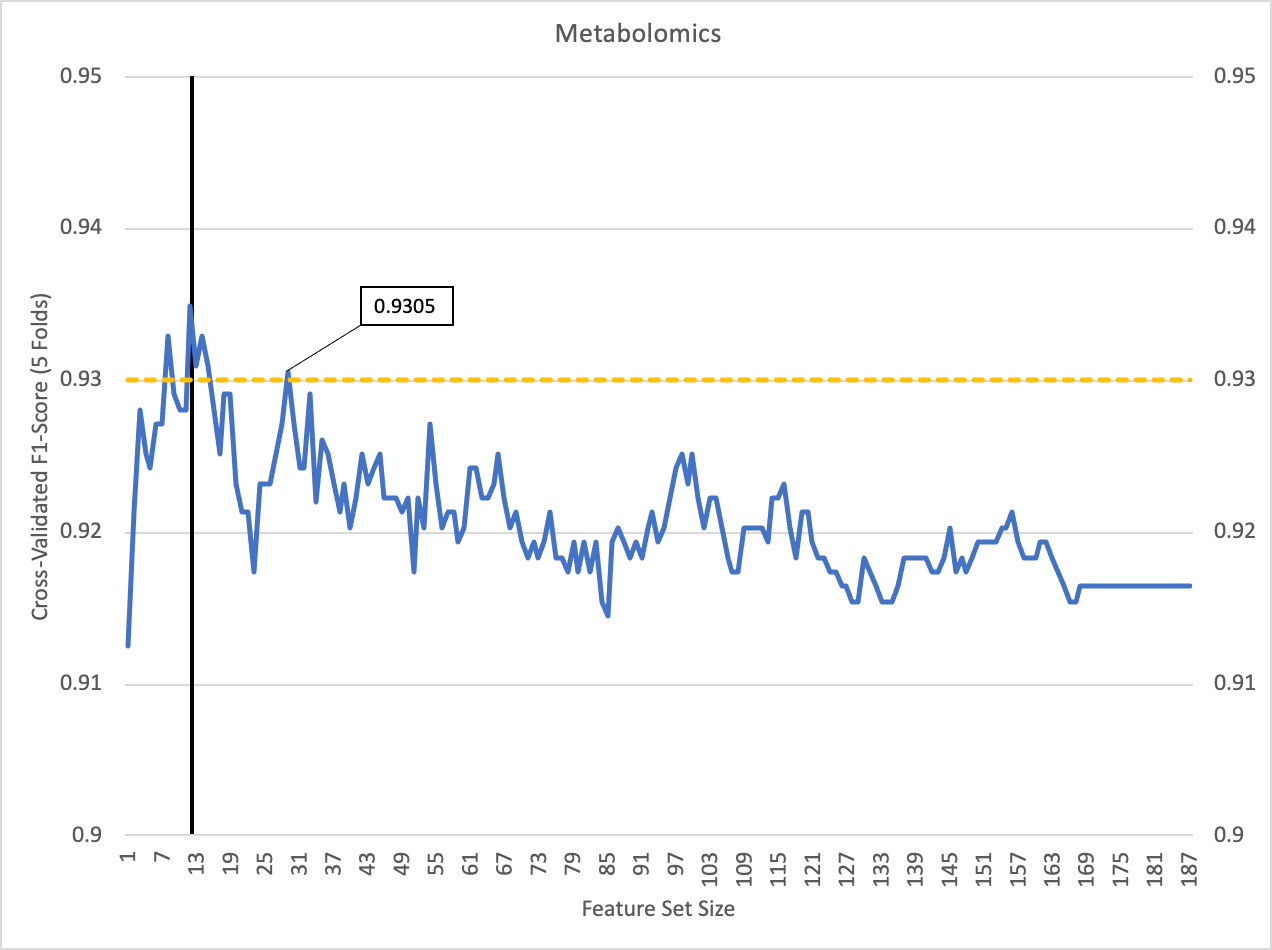


**S7a Figure: Interactions between genes for top transcripts.** Network constructing using GeneMANIA. Nodes represent genes, and nodes with stripes are genes represented in the transcriptomic list in Table 4. Edge colors represent different sources of associations between genes (*purple* – coexpression, *blue* – colocalization, *green* – genetic interactions). Genes without any connections are not included in the network graph.


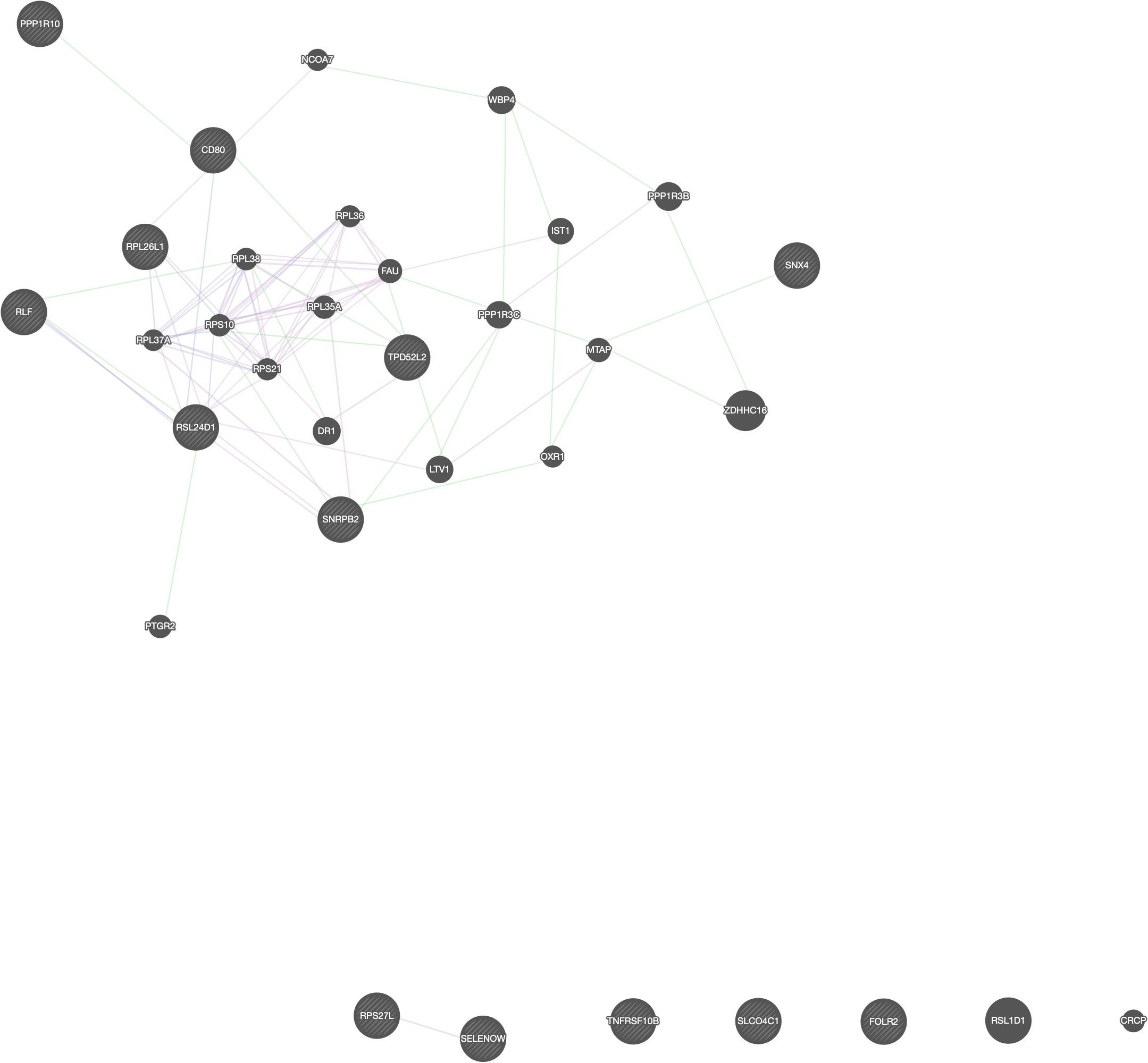


**S7b Figure: Interactions between top proteins.** Network constructed using GeneMANIA. Nodes repesent genes and nodes with stripes are genes in the proteomic list in Table 4. Edge colors represent different sources of associations between genes (*purple* – coexpression, *blue* – colocalization, *light blue* – pathway, *green* – genetic interactions, *yellow* – shared protein domains, *pink* – physical interactions). Proteins without any connections are not included in the network graph.


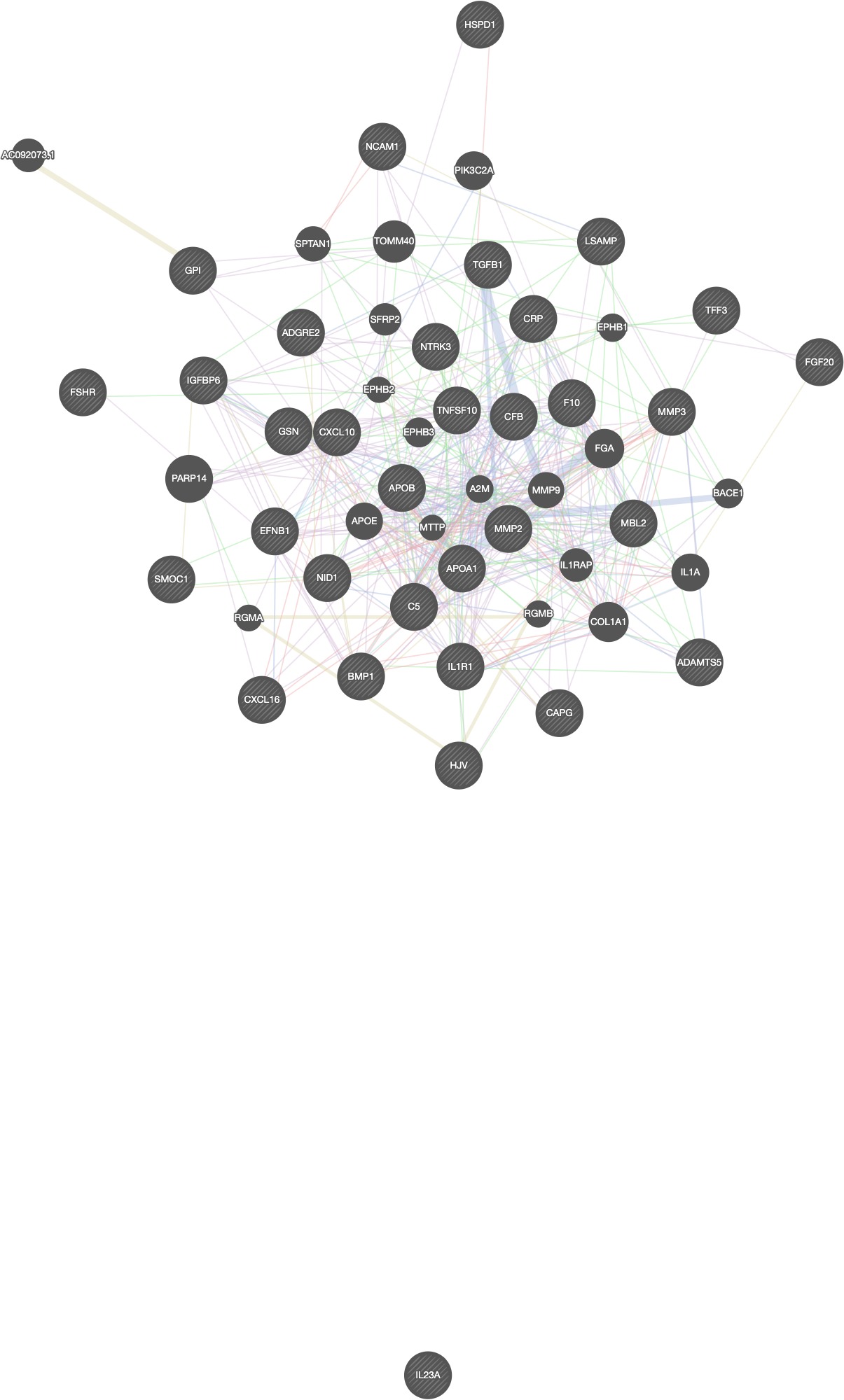


**S7c Figure: Interactions between top genes and proteins.** Network constructed using GeneMANIA. Nodes represent genes/proteins and nodes with stripes are genes/proteins in Table 4. Of the nodes with stripes in Table 4, the red circle represent genes from the transcriptomic list, all others are from the proteomic list. Edge colors represent different sources of associations between genes (*purple* – coexpression, *blue* – colocalization, *pink* – physical interactions, *green* – genetic interactions, *yellow* – shared protein domains). Genes and proteins without any connections are not included in the network graph.


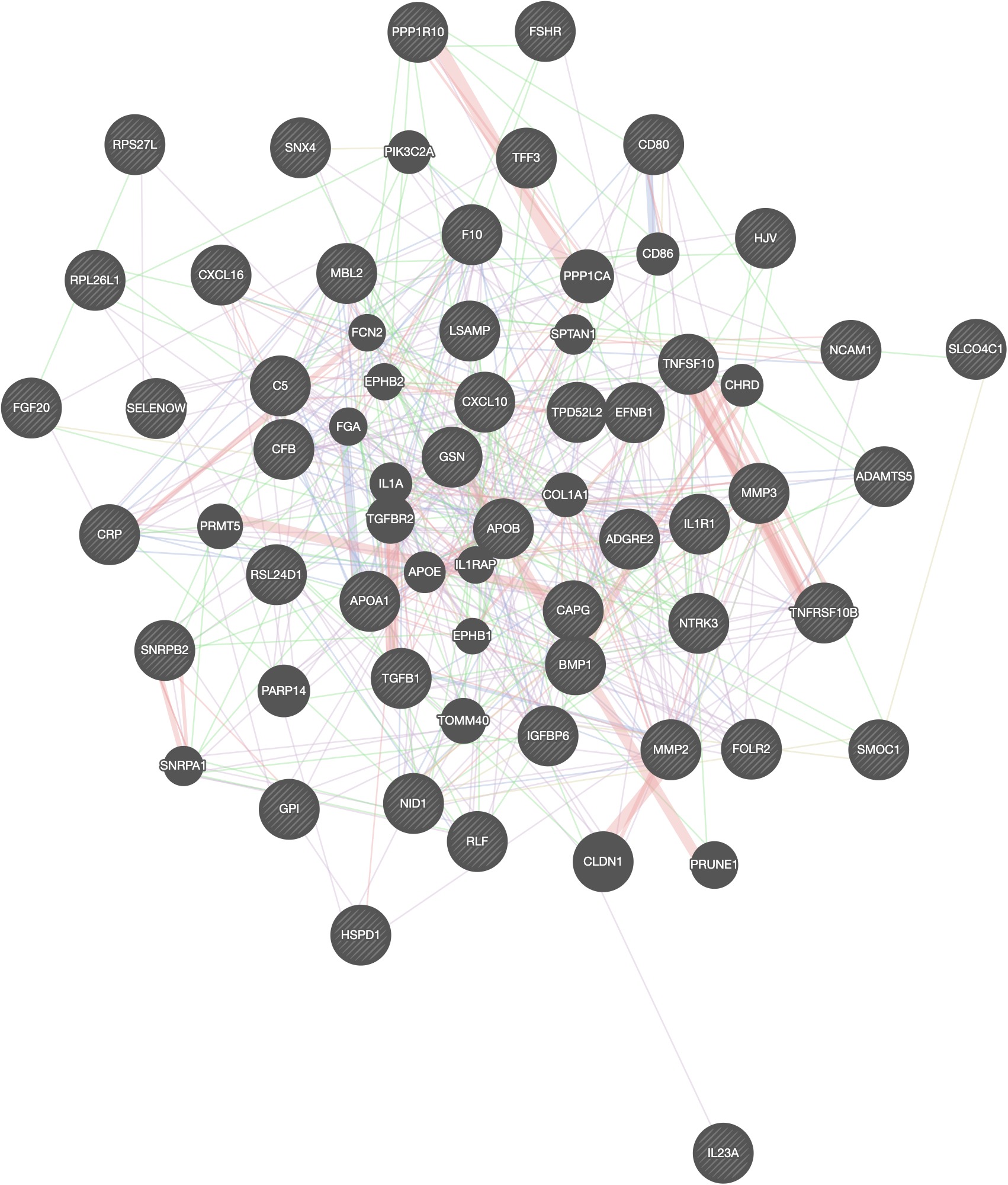

Supplement: S1 File — (DOCX) [file pone.0255337.s012.docx]
