# Supplementary material for: Multi-omics subtyping pipeline for chronic obstructive pulmonary disease
Source: PLoS One. 2021 Aug 25;16(8):e0255337. doi: 10.1371/journal.pone.0255337 (PMC8386883; doi:10.1371/journal.pone.0255337)
Supplement: S1 Appendix — (DOCX) [file pone.0255337.s013.docx]

**S1 Appendix: Supporting Methods and Results**

**Supporting Methods**

*Study Population Details:* The NIH sponsored multicenter Genetic Epidemiology of COPD (COPDGene, ClinicalTrials.gov Identifier: NCT01969344) study was approved and reviewed by the institutional review board at all participating centers (1). Written informed consent was received from all subjects. The COPDGene study enrolled 10,263 Non-Hispanic White (NHW) and African-American (AA) individuals from January 2008 until April 2011 (Phase 1) who were aged 45-80 with ≥10 pack-year smoking history and no exacerbations for >30 days. From the original cohort, 1,790 died and 1,567 were never reachable for any follow up. From July 2013 to July 2017, 6,758 subjects returned for an in-person 5-year visit at Phase 2. In addition, 457 age and gender matched healthy individuals with no history of smoking were enrolled as controls (mostly at Phase 2).

*Case definitions:* COPD was defined using spirometric evidence of airflow obstruction [post-bronchodilator forced expiratory volume at one second (FEV_1_)/forced vital capacity (FVC) <0.70], with severity defined as: mild or moderate (FEV_1_ ≥ 50% predicted) or severe (FEV_1_ < 50% predicted). Chronic bronchitis was defined as self-reported chronic cough and sputum for at least three months in each of the two years prior to baseline. Quantitative emphysema was quantified by percent of lung voxels -950 Hounsfield Units (% low attenuation areas: %LAA) on the full inspiratory CT scans. Visual emphysema was assessed as described by (2). Exacerbations were defined as acute worsening of respiratory symptoms requiring treatment with oral corticosteroids and/or antibiotics, emergency room visit, or hospital admission (3).

*Transcriptomics Details:* As described previously (4), total RNA was extracted from whole blood samples. The cDNA library was prepared with the Illumina TruSeq Stranded Total RNA with Ribo-Zero Globin kit. 75 bp paired end reads were generated in a HiSeq 2500 flow cell. Then the samples were sequenced to an average depth of 20 million reads. RNA reads were trimmed with Skewer software under default settings. The trimmed RNA reads were aligned to the GRCH38 genome. More technical details can be found in Parker et al’s previous study (4). We excluded 13 subjects from the final analysis for failing in quality control (QC) or absence of clinical phenotypes of interest. We only considered protein coding genes, and we filtered out genes with low expression (less than 10 counts total across samples). Post filtering, we obtained 2,637 samples and 18,487 genes. These filtered counts were then normalized using Trimmed Mean of M-values (TMM) to account for library size variation and corrected for batch effect and cell count covariates using a linear model. Finally, the corrected sequencing counts were transformed to be homoscedastic via Variance modeling at the observation-level (VOOM)(5).

*Proteomics Details:* The SomaLogic SOMAscan platform utilizes their single stranded DNA-base protein affinity method called SOMAmers (Slow Off-rate Modified Aptamers) to quantify protein levels in plasma(6). SOMAscan^Ⓡ^ multiplex proteomic assay is quantified using microarrays, and 1,017 SOMAmers were measured. SomaLogic conducted quality assurance on each sample; hybridization normalization to control for variability of microarrays; median normalization to control for total signal differences between samples, calibration to remove the variation between runs, and plate scaling to account for plate-by-plate variation using their recommended methods.

*Metabolomics Details:* The Metabolon platform reported 1,392 features. 1,064 annotated features were annotated and grouped: 436 lipids, 261 xenobiotics, 207 amino acids, 40 peptides, 38 cofactors and enzymes, 35 nucleotides, 25 carbohydrates, 11 energy pathway compounds, and 11 partially characterized molecules. A data normalization step was performed by Metabolon to correct variation resulting from instrument inter-day tuning differences: metabolite intensities were divided by the metabolite run day median then multiplied by the overall metabolite median. We determined that no further normalization was necessary based on the reduction in the significance of association between the top principal components and sample run day after normalization**.** Subjects with aggregate metabolite median z-scores greater than 3.5 standard deviation from the mean (N=6) of the cohort were removed. Metabolites were excluded if > 20% of samples were missing values (7) For the remaining 995 metabolites, missing values were imputed across metabolites with k-nearest neighbors imputation (k = 10) using the R package ‘impute’.

*Subject Filtering:* We removed subjects for the following reasons: 1) samples did not pass QC, 2) no phenotype data, 3) primary pulmonary diagnosis was not COPD, 4) never smoker, 5) reported having a lung transplant or lung volume reduction before the Phase 2 visit or 6) data processing as described for each of the -omics data sets. The final set of subjects was 2637, 1013 and 1057 for the transcriptomic, proteomic and metabolomic data sets respectively.

*Covariate Filtering Details:* The 242 clinical variables excluded blood cell counts, since the data had previously been adjusted for these counts, as well as age, sex, race, and BMI, to exclude features solely associated with demographic variables. To avoid bias due to outliers, non-parametric tests were used: either a Wilcoxon signed rank or Kruskal-Wallis based on whether the variable was continuous or categorical respectively. We accounted for multiple testing using the false discovery rate (FDR) procedure of Benjamini and Hochberg(8). Features remained if they were significantly associated with at least one clinical variable at a stringent threshold (within variable FDR adjusted p value < 10^-5^) to help optimize the efficiency of the dimension reduction step.

*Autoencoder (AE) Implementation Details*: The autoencoder used in this study was implemented using Tensorflow and Python 3.6. The number and size of layers in the encoder varies between datasets, with the larger transcriptomics model having five encoder layers of size 1024, 512, 128, 64 and a bottleneck layer of size 8, the metabolomics and proteomics data, having fewer features, was used to train a model of three layers of size 128, 64, 8. The bottleneck layer outputs the final embeddings used for clustering. While the sizes of layers are generally decreasing powers of two, some powers of two were omitted after they were observed to contribute little to model performance and to reduce the number of overall parameters to train. The loss function is the mean squared error, which is optimized by the adam optimizer(9). Training of the autoencoder was performed on 90% of the data with 10% being held out for testing.

Because the encoder and decoder networks consist of fully-connected neural network layers with a large number of parameters, special considerations were made for training. To reduce training time and improve accuracy, each layer of the encoder and its corresponding decoder pair was trained separately, with all layers trained together with a lower learning rate in a final “fine-tuning” step. To further reduce the number of trained parameters, and thus the training time and potential for overfitting, the decoder layer uses the transpose of the corresponding encoder weights so that only the encoder layers need to be trained. Also due to the number of total network parameters, a large number of passes through the dataset (epochs) was necessary to reach convergence in training loss. Generally, we used between 200-300 epochs per layer and 400 epochs in the finetuning- phase. The learning rates for the per-layer and finetuning- phases of training were 10^-4^ and 10^-6^, respectively.

*Dimension Reduction Details*:

Choosing the appropriate size of the encoder latent space involves a trade-off between using a less complex space, which will be more amenable to clustering with respect to scalability in number of dimensions, and a more efficient encoding, which will capture more of the intrinsic information in the dataset. Therefore, a mixture of cross-validation and silhouette score was used to choose the number of encoder output nodes (or PCA components), which directly relates to the size of the encoded latent space. Cross-validation is performed with a random 90/10 split of the subjects and repeated 3 times in order to generate an average value of Mean Squared Error (MSE) in the training and test splits. The sihouette score was calculated on the two subtypes based on running k-means with k=2 on each number of encoder output nodes.

The number of nodes was chosen near the “elbow” in the MSE curves with the maximum silhouette score as long as the average training error was nearly equal to the test error. If the silhouette score is too low, then the latent space may be too complex and therefore difficult to cluster, and if the testing error is too high compared to the training error, then the amount of information captured by the encoder is not enough to represent the data adequately. The chosen number of embeddings can be compared to the equivalent number of principal components by inverting both the embeddings and components to reconstruct the full data set (using the decoder and loadings, respectively) then measuring the MSE between the reconstructed data and the original. A lower MSE corresponds to a better reconstruction and suggests that the embeddings/components contain more information from the data used in the reconstruction.

*Clustering Details:* K-means was implemented in scikit-learn and we tried different values of k=[2-10]. MineClus(10) is an iterative subspace clustering algorithm that identifies the densest subspaces for a given dataset $D$, where rows are subjects and columns are dimensions (features), in a greedy manner as follows: at each iteration, MineClus selects $M=2/\alpha$ medoids from rows of $D$ at random. Then, for each $m\in M$, it finds $S_{m}$, which is the set of all dense dimensions around $m$ (i.e., the dimensions that have at least $min_{c}=\alpha\times|D|$points within a $w$-radius from $m$, where $|D|$ is the number of samples in the dataset, and $\alpha\in(0,1)$ is a user-defined parameter). These that determines the minimum size of desirable subspace dense dimensions are then combined to form dense subspaces. MineClus leverages frequent itemset mining (10) to avoid producing sparse subspaces while combining the dense dimensions. The dense subspace $s_{m}\subseteq S_{m}$ maximizing $\mu_{m}=c_{m}\times(1/\beta)^{|s_{m}|}$, where $c_{m}$ is the number of points that are within a $w$-range from $m$ in all dimensions in $s_{m}$, is chosen as the best subspace for $m$. Next, the medoid with the highest $\mu$score is selected as the best medoid at the current iteration. Then, a cluster including $c_{m}$ and $m$ is formed at $s_{m}$. Finally, $m$ and $c_{m}$ are removed from the dataset. This process is repeated until no more clusters can be found, i.e., there are fewer than $min_{c}$ points left in $D$, or none of the medioids have at least $min_{c}$ points in their w-distance neighborhood. We refer to the points that do not belong to any clusters as *outliers*.

To tune $w$, we first performed MineClus multiple times in the range of (0,40] with steps of 0.5 for $w$. Then we ran MineClus in the range that produced 2-4 clusters with steps of 0.05 for $w$. Finally, we implemented and examined a more inclusive version of MineClus, such that at each iteration “all” the remaining data points (as opposed to $2/\alpha$ random points) are tested as medoids to make sure all clustering options are considered We observed that this version had a higher stability (i.e., clusterings changed less as we varied $w$). However, it often resulted in clusterings with lower silhouette coefficients (described below) since MineClus is designed to maximize the number of dimensions as well as number of subjects in the clusters, rather than compactness of the clusters Hence, eventually we chose the original version of MineClus for our experiments. For the sake of reproducibility, we set the initial seed to 11 which is incremented by 13 at each iteration to avoid selecting the same medoids at all iterations. For each dataset, we picked the clustering with the highest silhouette coefficient under the condition that the silhouette coefficients for its individual clusterings were greater than 0.10. For some of the integrated or stratified analyses, there was no clustering such that all the clusters had silhouette > 0.10. In those cases, we picked the one with the highest overall silhouette.

*Metrics:* The silhouette coefficient measures the quality of a clustering (11) based on the cohesion and separation of its clusters. The silhouette coefficient for each point $p$ is calculated as $sil_{p}=\frac{a-b}{max(a,b)}$, where $a$ is the average distance between $p$ and the points in the same cluster, while $b$ shows the average distance between $p$ and all the points in the nearest cluster. The silhouette score for a clustering $C$ is calculated as $sil_{C}=\frac{\sum_{i=1}^{|D|} sil_{p_{i}}}{|D|}$, which is a normalized value in [−1,+1], where +1 indicates perfect clustering (i.e., points in the same clusters are close to each other and points in different clusters are relatively far from each other), while -1 indicates the reverse. The connectedness score(12) evaluates the compactness of the clusterings. The connectedness of each point $p$ is defined as the ratio of $m$-nearest neighbors of $p$ that are in the same cluster as $p$. The connectedness for a clustering is computed by averaging the connectedness values of all points, which is between [0,1]. 1 means for each point all of its $m$-nearest neighbors are in the same cluster, while 0 indicates the opposite. Connectedness is biased towards fewer clusters, as increasing the number of clusters increases the chance for the closest neighbors to end up in different clusters. We used $m=10$ for the experiments, which is suggested as the default value in the cIValid R package(13).

*Sensitivity Analysis:* The gap statistic introduced by Tibshirani et al., (14) is an alternative for the elbow method that is widely used to determine the optimal number of clusters $k$. For each $k$, the intra-cluster distance ${IC}_{in,k}(D)$, which is the sum of squared Euclidean distances between each pair of points in the same cluster, is computed. Then the same clustering algorithm is executed with $R$ datasets. Each dataset in $R$ has the same number of subjects and dimensions as $D$. The values of each dimension are generated using a Gaussian distribution with the same mean and variance as the corresponding dimension in $D$. Then ${IC}_{in,k}(R)$, which is the average of intra-cluster distances for the $R$clusterings, is computed. The gap value of $k$ is computed as $gap(k)={IC}_{in,k}(R)-{IC}_{in,k}(D)$. The smallest value of $k$ such that the $gap(k)$ is within one standard deviation of the $gap(k+1)$, i.e., the smallest $k$ such that $\Delta(gap)=gap(k)-(gap(k+1)-SD(gap(k+1))>0$, where $SD(gap(k+1))$ is the standard deviation of intra-cluster distances for the $R$ clusterings with $k$ clusters, is selected as the optimal $k$.

In addition to gap statistic, we used the Jaccard similarity for evaluation of clustering stability. This measure is commonly used to measure the similarity between two sets A and B and defined as $\frac{|A\bigcap B|}{|A\cup B|}$, where the numerator is the number of of items that both sets have in common, and the denominator indicates the number of all unique items in both sets. However, when the cardinality of two sets is very different, the Jaccard similarity becomes small, even if the larger set contains all the elements of the smaller set. In order to address the sensitivity of Jaccard to the cardinality of the sets, we suggest using the normalized Jaccard, which is defined as $\frac{|A\bigcap B|}{|\min( \left| A \right|, \left| B \right|)}$ where now the denominator is the cardinality of the smaller set, and is the maximum number of items that two sets can have in common. To compute the Jaccard similarity between two clusterings *C=(c_1_,…, c_k_)* and *C’=(c’_1_,…, c’_k’_)*, where $k\leq k'$, we generate all the possible mappings from $C$ to $C'$, where a mapping $C\to C'=(m_{1},...,m_{k})$ and each ${m_{x}}=(c_{y},{c'}_{z})$ maps each cluster of $C$ to exactly one cluster of $C'$. For each mapping, we compute the Jaccard coefficient as $J_{M(C,C')}=\frac{\sum_{i=1}^{k} \cup m_{i}}{min(|C|,|C'|)}$, where $\cup m_{i}$shows the number of subjects that exists in both clusters in $m_{i}$. Finally, we select the mapping that has the largest Jaccard coefficient as the best mapping. The Jaccard coefficient is between 0 and 1, where 1 indicates absolute similarity and 0 indicates no similarity.

We used the Jaccard coefficient for two purposes: parameter tuning and measuring the stability of the clusterings. To tune $\alpha$ and $\beta$, we ran MineClus with synthetic datasets and observed that the mined clusterings with $\alpha$≃0.1 and $\beta\simeq0.25$ had the highest Jaccard coefficient with the actual clusterings (data not shown), which is on par with the suggested values for $\alpha$ and $\beta$in (10).

We took a similar approach to (15) in order to measure the stability of the clustering at each $w$. To this end, for each input dataset $D$, we generated 20 sampled datasets (${D'}_{1},...,{D'}_{20}$) with the sampling rate of 80% and without replacement. We then identified $W_{k=2}$ which is the set of $w$’s with which MineClus finds two clusters in $D$. Next, for each $w\in W_{k=2}$, we ran MineClus with the 20 sampled datasets and defined the stability of $w$ as $\frac{\sum_{i=1}^{20} J(C_{D,w}, C_{{D'}_{i},w})}{20}$, where $C_{x,y}$ shows the clustering of dataset $x$ for $y=w$, and $J(C_{i},{{C_{j}})}$shows the Jaccard coefficient between the two clusterings $C_{i}$and $C_{j}$. The resulting stability score is between 0 and 1, where 1 indicates perfect stability and 0 indicates no stability.

A final metric we considered was the consistency of membership of subjects in clusters. As the mined clusterings are sensitive to the random medioids, we observed that the subjects (especially the subjects in the smaller cluster) migrate from one cluster to another at each $w$. Therefore, for each subject we computed its membership percentage in the clusterings with 2 clusters. In **S5 Figure**, the membership percentages to the larger/smaller/outliers are mapped to a red-green-blue (RGB) color tuple. For example, if there are 10 clusterings, and Subject #1 was in the outlier/larger/smaller clusters 2/5/3 times, the RGB tuple will be (0.2, 0.5, 0.3), which is mapped to
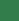
. Or if Subject #2 was in the outlier/larger/smaller clusters 7/2/1 times, the corresponding RGB tuple will be (0.7, 0.2, 0.1), which is mapped to
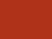
.

*Feature Selection Details:* SVMRFE was implemented using recursive feature elimination with cross validated selection (RFECV) method from scikit-learn as the feature selection method and the LinearSVC method as the SVM. RFECV performs cross-validation on top of standard recursive feature elimination in order to choose the best number of features from the highest scoring number of features across each fold. Cross-validation was implemented using the Stratified K-Fold method from scikit-learn with the number of folds set to five. Sets of features (in increasing size from 1 to p features) can be ranked based on the f1 classification scoring metric(16), averaged over each fold of validation. This rank-ordered list of feature sets can be used to select the optimal set of features by choosing the feature set with the highest average metric.

*Annotations for Enrichment Analysis:* The transcriptomics and proteomics datasets used the GeneOntology annotations provided by PANTHER(17) for molecular function and biological process. Identifiers made available from the SOMAscan platform for each protein quantified were used to annotate the proteomics data. Using the entire annotation provided by PANTHER’s annotation service, which includes all levels of the annotation hierarchy, a separate p-value adjustment was applied to each level individually as well as to the entire hierarchy. Metabolomic data was annotated using pathways provided by Metabolon. The background size of each pathway/ontology for each -omic dataset was determined from the number of features pre-covariate filtering. Ontologies and pathways with FDR < 0.10 (relative to level) are reported in the manuscript.

**Supporting Results**

*Sensitivity Analysis:* We performed three sensitivity analyses to evaluate the robustness of our approach, and selection of tuning parameters *k* and *w*. First, for the number of clusters *k*, we used the gap-statistic to explore whether clustering is supported by the data (*k*= 1 compared to *k*> 1). The gap statistic indicates *k*> 1 for both Transcriptomic and Metabolomic data (but we use the results and our internal metrics to select amongst *k*> 1), but *k*= 1 for the Proteomic data indicating a weaker clustering signal for that data set (**S2 Figure**). Then, we explored the effect of changing the MineClus tuning parameter *w* in two ways. First, by clustering re-sampled data using the same procedure, the new cluster assignments were stable and had high overlap with the original clusters for different values of *w* (**S3 Figure**). Third, on the original data (not resampled), we also examined the consistency of large and small subtype assignments (membership stability) for each subject for different values of *w*. For all three data sets, the large subtype tends to be very stable regardless of *w* (i.e., subjects tend to be consistently assigned to the large subtype). The small subtype and outlier subjects show more variability in their assignment, but generally are stable for the transcriptomics and metabolomics data. Occasionally the small cluster subjects are assigned as outliers for the transcriptomic data (purple points in **S4 Figure**).

**References**

1. Regan EA, Hokanson JE, Murphy JR, Make B, Lynch DA, Beaty TH, et al. Genetic epidemiology of COPD (COPDGene) study design. COPD. 2010;7(1):32-43.

2. Lynch DA, Moore CM, Wilson C, Nevrekar D, Jennermann T, Humphries SM, et al. CT-based Visual Classification of Emphysema: Association with Mortality in the COPDGene Study. Radiology. 2018;288(3):859-66.

3. Bowler RP, Kim V, Regan E, Williams AAA, Santorico SA, Make BJ, et al. Prediction of acute respiratory disease in current and former smokers with and without COPD. Chest. 2014;146(4):941-50.

4. Parker MM, Chase RP, Lamb A, Reyes A, Saferali A, Yun JH, et al. RNA sequencing identifies novel non-coding RNA and exon-specific effects associated with cigarette smoking. BMC Med Genomics. 2017;10(1):58.

5. Law CW, Chen Y, Shi W, Smyth GK. voom: Precision weights unlock linear model analysis tools for RNA-seq read counts. Genome Biol. 2014;15(2):R29.

6. Gold L, Ayers D, Bertino J, Bock C, Bock A, Brody EN, et al. Aptamer-based multiplexed proteomic technology for biomarker discovery. PLoS One. 2010;5(12):e15004.

7. Bijlsma S, Bobeldijk I, Verheij ER, Ramaker R, Kochhar S, Macdonald IA, et al. Large-scale human metabolomics studies: a strategy for data (pre-) processing and validation. Anal Chem. 2006;78(2):567-74.

8. Benjamini Y, Hochberg Y. Controlling the False Discovery Rate: A Practical and Powerful Approach to Multiple Testing. Journal of the Royal Statistical Society Series B (Methodological). 1995;57(1):289-300.

9. Kingma DPJB. Adam: A Method for Stochastic Optimization. 2014.

10. Man Lung Y, Nikos M, editors. Frequent-pattern based iterative projected clustering. Third IEEE International Conference on Data Mining; 2003 22-22 Nov. 2003.

11. Rousseeuw PJ. Silhouettes: A graphical aid to the interpretation and validation of cluster analysis. Journal of Computational and Applied Mathematics. 1987;20:53-65.

12. Handl J, Knowles J, Kell DB. Computational cluster validation in post-genomic data analysis. Bioinformatics. 2005;21(15):3201-12.

13. Brock G, Pihur V, Datta S, Datta S. clValid: An R Package for Cluster Validation. 2008. 2008;25(4):22.

14. Tibshirani R, Walther G, Hastie T. Estimating the number of clusters in a data set via the gap statistic. Journal of the Royal Statistical Society: Series B (Statistical Methodology). 2001;63(2):411-23.

15. Hennig C. Cluster-wise assessment of cluster stability. Comput Stat Data Anal. 2007;52:258-71.

16. William BF, Ricardo B-Y. Information retrieval: data structures and algorithms: Prentice-Hall, Inc.; 1992.

17. Mi H, Muruganujan A, Ebert D, Huang X, Thomas PD. PANTHER version 14: more genomes, a new PANTHER GO-slim and improvements in enrichment analysis tools. Nucleic Acids Res. 2019;47(D1):D419-D26.
